# Engineered Exosomal miR‐146a‐5p Reprograms BMSC Fate and Restores Mitochondrial Homeostasis in Glucocorticoid‐Induced Osteonecrosis of Femoral Head

**DOI:** 10.1002/advs.75897

**Published:** 2026-06-15

**Authors:** Zehui Lv, Xuejie Cai, Yiming Xu, Xingdong Yang, Ruoying Wang, Han Wang, Yixin Bian, Jiawei Xu, Jiao Lu, Lulu Liu, Yingjie Wang, Jibin Song, Bin Feng, Xisheng Weng

**Affiliations:** ^1^ Department of Orthopedic Surgery State Key Laboratory of Complex Severe and Rare Diseases Peking Union Medical College Hospital Chinese Academy of Medical Science and Peking Union Medical College Beijing China; ^2^ Biomedical engineering facility of National Infrastructures for Translational Medicine State Key Laboratory of Complex Severe and Rare Diseases in Peking Union Medical College Hospital Chinese Academy of Medical Science and Peking Union Medical College Beijing China; ^3^ State Key Laboratory of Chemical Resource Engineering College of Chemistry Beijing University of Chemical Technology Beijing P. R. China

**Keywords:** exosomes, glucocorticoid, miR‐146a‐5p, mitochondrial homeostasis, mitophagy, osteonecrosis of the femoral head, stem cell senescence

## Abstract

Glucocorticoid (GC)‐induced osteonecrosis of the femoral head (ONFH) involves stem cell senescence, mitochondrial dysfunction, and impaired bone regeneration. However, the molecular basis linking GC stress to bone marrow stromal cell (BMSC) dysfunction remains unclear. Here, we identify miR‐146a‐5p as a key regulator of BMSC fate under GC exposure, through comprehensive transcriptomic analysis of clinical bone marrow samples from GC‐induced ONFH patients. Exosomes engineered to deliver miR‐146a‐5p restored mitochondrial membrane potential, suppressed oxidative stress, and reactivated mitophagy by targeting the TRAF6‐NF‐κB axis. These exosomes reversed GC‐induced senescence and enhanced osteogenic and angiogenic capacity in vitro and in vivo. In a rat ONFH model, intraosseous delivery of miR‐146a‐5p exosomes improved trabecular structure and vascularization. Single‐cell RNA‐seq revealed a shift toward osteogenic and immunomodulatory BMSC subtypes. Our findings demonstrate that miR‐146a‐5p‐engineered exosomes rejuvenate skeletal regeneration by restoring mitochondrial homeostasis and inflammatory balance, offering a promising cell‐free therapy for GC‐associated ONFH.

## Graphical abstract

Exosomal miR‐146a‐5p is identified as a pivotal regulator in steroid‐induced osteonecrosis. Its reduction activates NF‐κB signaling, compromises mitophagy, and disrupts mitochondrial bioenergetics, resulting in autophagic disequilibrium. Engineered exosomes delivering miR‐146a‐5p reinstate mitochondrial function, augment oxidative phosphorylation and ATP generation, and reestablish autophagic flux in vivo, thereby promoting cellular homeostasis and functional recovery.

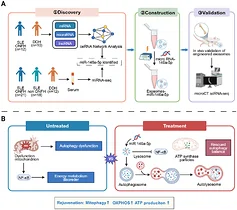

## 1 Introduction

Glucocorticoid (GC)‐induced osteonecrosis of the femoral head (ONFH) is a devastating orthopaedic condition characterized by ischemic bone death and collapse of the femoral head [1, 2, 3, 4]. High‐dose or prolonged glucocorticoid therapy is a leading cause of non‐traumatic ONFH [5], with an alarmingly high collapse rate (>60% within 1 year) in the absence of early intervention [6, 7]. Despite its prevalence, the precise molecular pathogenesis of GC‐induced ONFH remains incompletely understood, which hampers the development of effective targeted therapies. A fundamental barrier to regeneration in ONFH is the failure to repair necrotic bone, partly due to a compromised bone marrow niche [8]. In particular, mounting evidence has indicated that bone marrow mesenchymal stem cells (BMSCs), the progenitors for osteoblasts required in bone renewal, are functionally impaired in ONFH lesions [9, 10]. Physiological levels of GC could enhance BMSC osteogenesis, while excessive levels disrupt BMSC homeostasis, diminishing their proliferation and osteogenic differentiation capacity [11, 12]. Additionally, GC skew BMSC fate toward adipogenic lineage, contributing to marrow fat accumulation at the expense of new bone formation [11, 13]. The imbalance between osteogenesis and adipogenesis in BMSCs is now recognized as a primary pathological feature of GC‐associated ONFH. Moreover, GC exposure accelerates BMSC senescence, further impairing their regenerative functions and exacerbating bone loss. Together, these deficits create a local “regeneration obstacle” in which the endogenous repair capacity of the femoral head is severely blunted [9, 10, 14, 15].

Mechanistically, the deleterious effects of GC on bone cells involve a complex interplay between mitochondrial dysfunction, oxidative stress, and aberrant inflammatory signaling [16]. GC at high doses induces excess production of reactive oxygen species (ROS) in osteogenic cells, triggering oxidative damage and cell death via apoptosis, ferroptosis, and autophagy‐dependent pathways [17, 18, 19]. Mitochondria have emerged as central mediators in this process, serving both as a major source of GC‐induced ROS and as a critical target of oxidative injury [16, 17, 18]. Prolonged GC exposure overwhelms the mitochondrial antioxidant defenses, leading to loss of mitochondrial membrane integrity and collapse in ATP production [20, 21]. As mitochondrial homeostasis is compromised, a vicious cycle ensues whereby dysfunctional mitochondria release more ROS, which in turn inflicts further mitochondrial damage. Beyond bioenergetics, damaged mitochondria can precipitate inflammatory pathways by releasing danger signals such as mitochondrial DNA and ROS that activate the NLRP3 inflammasome and other stress response mechanisms [21, 22]. In ONFH, the death of bone and marrow cells under GC stress is associated with an abnormal osteoimmune response, creating a chronic inflammatory microenvironment that further impedes bone regeneration [12, 15, 23, 24]. This cross‐talk between mitochondrial dysfunction and inflammation amplifies tissue injury in a feed‐forward manner. Concurrently, autophagy, an intrinsic mechanism of cells for removing damaged organelles, is dysregulated by GC [19, 24]. Moderate activation of autophagy can be cytoprotective under stress; however, excessive or impaired autophagy has been observed in GC‐treated BMSCs. Indeed, excessive GC use is reported to cause autophagy disorders in bone cells. Inefficient autophagic clearance of dysfunctional mitochondria leads to their accumulation, exacerbating oxidative stress and inflammatory signaling [25]. On the other hand, excessive autophagy may directly contribute to cell death [26]. Thus, GC‐induced perturbations in mitochondrial function and autophagy jointly drive BMSC dysfunction and the sterile inflammation in ONFH lesions. These insights highlight a pathogenic triad of mitochondrial injury, autophagy imbalance, and inflammation underlying the regeneration failure in GC‐induced ONFH.

Amidst these mechanistic advances, there is growing interest in microRNA (miRNA)‐mediated regulation as a means of modulating the pathological pathways in ONFH [27, 28, 29]. MiRNAs are small noncoding RNAs that fine‐tune gene expression and are known to play crucial roles in both inflammatory responses and bone metabolism. In particular, miR‐146a‐5p has garnered attention due to its dual function in controlling inflammation and osteogenic activity [30]. miR‐146a is an NF‐κB‐responsive miRNA that acts as a negative feedback regulator of inflammation by directly targeting TRAF6 and IRAK1, key adaptor proteins in the NF‐κB pathway, thereby dampening the production of pro‐inflammatory cytokines [31, 32, 33, 34]. Under this mechanism, miR‐146a‐5p serves as an important brake on excessive NF‐κB signaling and has broadly been implicated in immunoregulation and osteoimmunology. Notably, miR‐146a also influences bone cell functions and has been linked to osteoblast differentiation and age‐related bone remodeling [30, 35, 36]. In the context of ONFH, miR‐146a‐5p appears to be a particularly relevant molecular switch. However, the precise regulatory network, including the potential role of miR‐146a‐5p in BMSC dysfunction and ONFH progression in human‐mediated mechanisms, remains poorly defined. To address this gap, we performed integrated multi‐omics profiling, including transcriptome‐wide messenger RNA (mRNA), miRNA, and long non‐coding RNA (lncRNA) sequencing on bone marrow samples from patients with GC‐induced ONFH undergoing total hip arthroplasty. Our analysis uncovered a disease‐associated miRNA signature, with miR‐146a‐5p emerging as a nodal regulator that links inflammation, senescence, and impaired osteogenesis within the Glucocorticoid‐damaged bone marrow niche. These findings set the stage for further mechanistic and therapeutic exploration.

**SCHEME 1:**
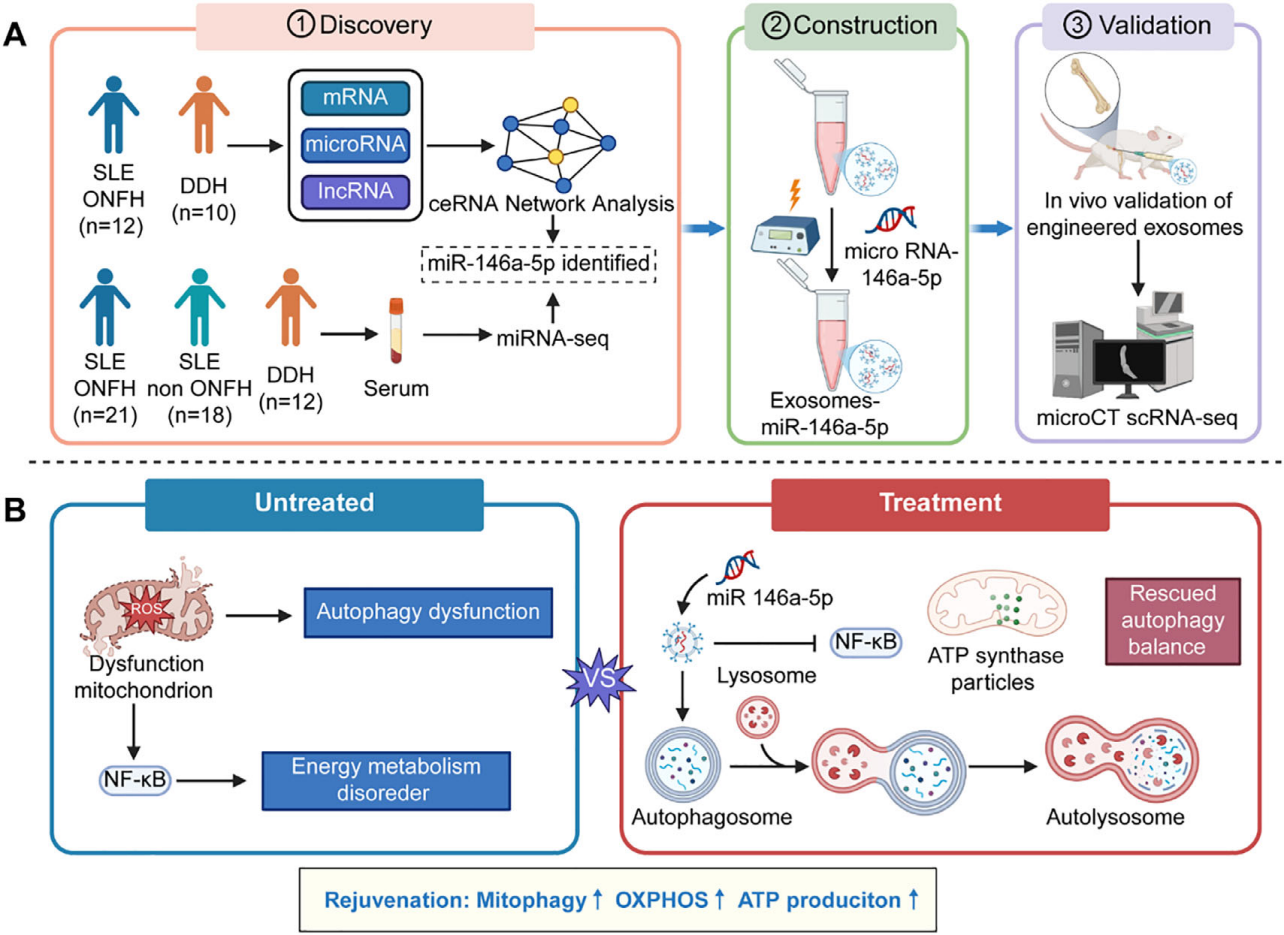
Schematic overview of the study design, experimental workflow and mechanistic insight into miR‐146a‐5p‐based exosomal therapy for GC‐induced ONFH.

In light of these considerations, we hypothesized that augmenting miR‐146a‐5p levels in GC‐damaged bone marrow niche might rejuvenate BMSC function and promote bone repair in ONFH. We were particularly inspired by emerging studies showing that exosome‐based delivery of regulatory miRNAs can effectively modulate cellular pathways in bone disease models. Exosomes are nano‐sized vesicles capable of shuttling miRNAs and other biomolecules to target cells, offering a promising vehicle for cell‐specific genetic modulation [37]. In this study, we leveraged BMSC‐derived exosomes as a miR‐146a‐5p delivery system to test their therapeutic potential in GC‐induced ONFH. We demonstrated that exosome transfer of miR‐146a‐5p markedly rescues BMSC functionality under GC stress. Treated BMSCs showed restoration of mitochondrial membrane potential and oxidative phosphorylation, normalization of autophagic flux, and a reduction in pro‐inflammatory signaling activity compared to untreated GC‐injured controls. Furthermore, miR‐146a‐5p‐enriched exosomes enhanced the osteogenic differentiation capacity of BMSCs and improved bone regeneration in a GC‐induced ONFH animal model. These findings underscore a previously unappreciated mechanism whereby miR‐146a‐5p governs the crosstalk between mitochondrial quality control, autophagy, and inflammation in BMSCs. Our work revealed a novel therapeutic avenue of miR‐146a‐5p restoration via exosomal delivery to protect the bone regenerative niche from GC‐induced damage, offering a promising strategy to mitigate GC‐induced ONFH progression. A schematic overview of the study design, experimental workflow, and proposed mechanism is presented in Scheme 1.

## 2 Results

### 2.1 Integrated Multi‐Omics Profiling Identifies miR‐146a‐5p as a Core Regulator Linking Inflammation, Aging, and Impaired Osteogenesis in SLE‐Associated Osteonecrosis

**FIGURE 1:**
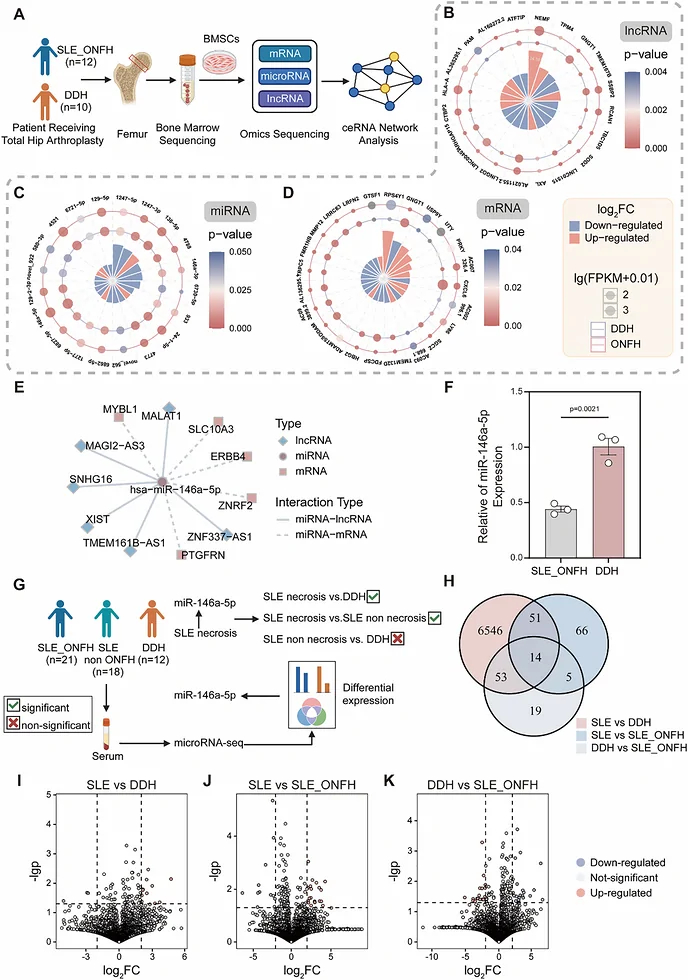
Integrated multi‐omics analysis identifies miR‐146a‐5p as a core regulatory node in SLE‐associated osteonecrosis of the femoral head. (A) Schematic of study design. BMSCs were isolated from patients with SLE‐ONFH (n = 12) and DDH (n = 10) undergoing total hip arthroplasty. Whole‐transcriptome sequencing was performed for mRNAs, miRNAs and lncRNAs profiling, followed by ceRNA network analysis. BMSCs, bone marrow mesenchymal stem cells; SLE‐ONFH, systemic lupus erythematosus‐associated osteonecrosis of the femoral head; DDH, developmental dysplasia of the hip; mRNAs, messenger RNAs; miRNAs, microRNAs; lncRNAs, long non‐coding RNAs; ceRNA, competing endogenous RNA. (B–D) Radar plots showing differential expression profiles of lncRNAs (B), miRNAs (C) and mRNAs (D) between ONFH and DDH‐derived BMSCs. Node size reflected expression abundance and color indicated up‐ or downregulation. (E) CeRNA network analysis positions miR‐146a‐5p as a central node interacting with key osteogenesis‐ and inflammation‐related lncRNAs and mRNAs. (F) RT‐qPCR validation of miR‐146a‐5p expression in an independent cohort of BMSCs. RT‐qPCR, quantitative real‐time polymerase chain reaction (n = 3). (G) Study designed for serum miRNAs profiling. Circulating miRNAs were analyzed in SLE‐ONFH (n = 21), SLE‐non ONFH (n = 18) and DDH (n = 12) patients to evaluate necrosis‐specific signatures. (H) Venn diagram showing the overlap of differentially expressed circulating miRNAs among SLE‐ONFH vs. DDH, SLE‐ONFH vs. SLE‐non ONFH and DDH vs. SLE‐non ONFH comparisons. (I–K) Volcano plots depicting differential miRNAs expression between groups. MiR‐146a‐5p is consistently identified as significantly downregulated in SLE‐ONFH. Data are presented as mean ± SEM. Two‐tailed Student's t‐test was used for comparison. Exact P values are reported in the figure.

To dissect the molecular basis underlying BMSC dysfunction in GC‐induced ONFH, we first performed comprehensive transcriptomic profiling of BMSCs isolated from patients with systemic lupus erythematosus (SLE)‐associated ONFH and non‐inflammatory controls with developmental dysplasia of the hip (DDH) undergoing total hip arthroplasty (Figure 1A). BMSCs were isolated and purified, and then verified by flow cytometry using three positive (CD44, CD73, and CD90) and three negative markers (CD34, CD45, and HLA‐DR) as in our previous report [38]. Whole‐transcriptome sequencing encompassing lncRNAs, miRNAs, and mRNAs revealed widespread and coordinated alterations in gene expression programs (Figure 1B‐D).

Among dysregulated non‐coding RNAs, miR‐146a‐5p emerged as one of the most significantly and consistently downregulated miRNAs in ONFH‐derived BMSCs (Figure 1B). Notably, network analysis of competing endogenous RNA (ceRNA) interactions positioned miR‐146a‐5p at the hub of a dysregulated regulatory axis, bridging multiple osteogenic and inflammatory mediators, including lncRNAs such as XIST and MALAT1, and mRNAs like PTGFRN and ZNRF2 (Figure 1E). These findings suggested that loss of miR‐146a‐5p may orchestrate transcriptomic rewiring under GC‐induced stress conditions, impacting both inflammation and differentiation pathways. To validate these observations, we performed quantitative real‐time polymerase chain reaction (RT‐qPCR) in an independent cohort and confirmed significant downregulation of miR‐146a‐5p in ONFH‐BMSCs compared to DDH‐BMSCs (Figure 1F).

Next, to determine whether miR‐146a‐5p dysregulation also extends into the systemic circulation, we performed serum miRNA sequencing in an expanded cohort comprising SLE‐ONFH, SLE‐non‐ONFH, and DDH patients (Figure 1G). Strikingly, 14 miRNAs were specifically decreased in the SLE‐ONFH group but remained unchanged between SLE‐non‐ONFH and DDH individuals, suggesting a necrosis‐specific molecular signature independent of underlying SLE activity (Figure 1H, Table S1). Further intersectional analysis of differentially expressed circulating miRNAs across the three groups revealed miR‐146a‐5p as the only molecule consistently altered, both in bone marrow cells and in circulation (Figure 1I‐K). This dual‐compartment alteration strengthens its candidacy as a mechanistic regulator and a readily accessible circulating biomarker associated with ONFH. To further support a glucocorticoid‐responsive regulation of miR‐146a‐5p, BMSCs were exposed to increasing concentrations of methylprednisolone (MPS) in vitro, which resulted in a dose‐dependent decrease in miR‐146a‐5p expression (Figure S1).

Our integrative multi‐omics analysis identified miR‐146a‐5p as a core noncoding RNA hub that links inflammatory signaling, senescence, and impaired osteogenesis in SLE‐associated ONFH. Its consistent downregulation in both BMSCs and serum highlighted miR‐146a‐5p as a promising molecular target for diagnostic evaluation and therapeutic intervention in ONFH.

### 2.2 Exosomes Depletion of miR‐146a‐5p Drives BMSCs Senescence and Lineage Imbalance in ONFH

**FIGURE 2:**
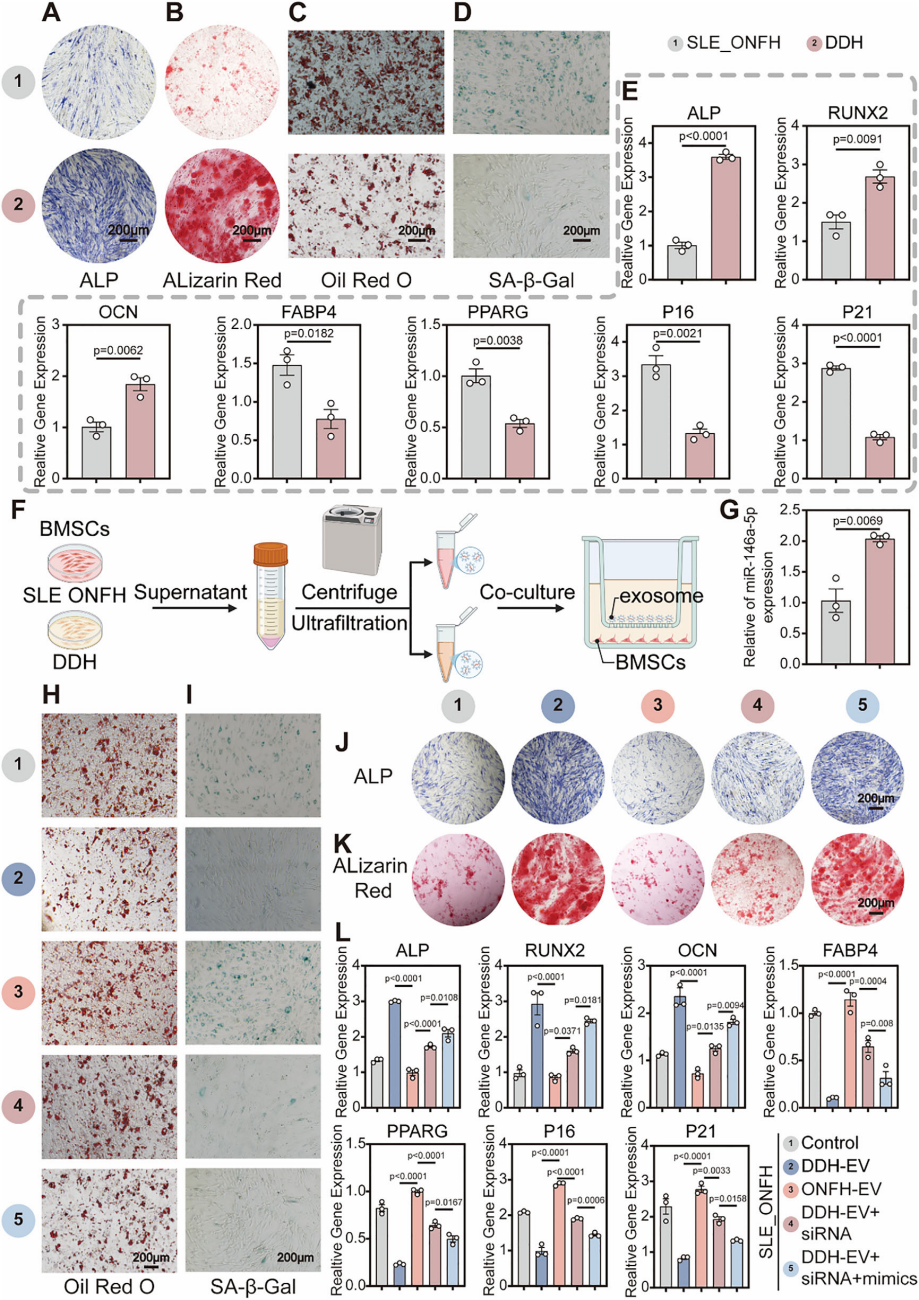
Exosome downregulation of miR‐146a‐5p promoted BMSCs senescence and lineage imbalance in ONFH. (A‐D) Phenotypic assays of BMSCs derived from SLE‐ONFH and DDH patients. Representative images of ALP staining, Alizarin Red staining, Oil Red O staining and SA‐β‐gal staining are shown, indicating impaired osteogenesis, enhanced adipogenesis and elevated senescence in ONFH‐BMSCs. ALP, alkaline phosphatase; SA‐β‐gal, senescence‐associated β‐galactosidase. (E) RT‐qPCR analysis of osteogenic (ALP, RUNX2 and OCN), adipogenic (FABP4 and PPARG) and senescence (P16 and P21) markers in SLE‐ONFH versus DDH BMSCs (n = 3). (F) Schematic diagram showing exosome isolation and co‐culture setup. BMSCs were cultured; conditioned media were collected, exosomes were purified via ultracentrifugation and then applied to recipient BMSCs in transwell co‐culture. (G) RT‐qPCR quantification of miR‐146a‐5p expression levels in exosomes derived from SLE‐ONFH and DDH BMSCs (n = 3). (H and I) Functional rescue assays. Oil Red O and SA‐β‐gal staining of BMSCs co‐cultured with different exosome treatments including control, DDH‐derived exosomes, ONFH‐derived exosomes, ONFH‐exosomes with miR‐146a‐5p knockdown (siRNA) and ONFH‐exosomes with miR‐146a‐5p supplementation (mimic). (J and K) ALP and Alizarin Red staining showing osteogenic differentiation after exosome treatments. (L) RT‐qPCR analysis of osteogenic (ALP, RUNX2 and OCN), adipogenic (FABP4 and PPARG) and senescence (P16 and P21) gene expression across treatment groups (n = 3). Data are presented as mean ± SEM. Two‐tailed Student's t‐test or one‐way ANOVA with Tukey's post hoc test was used for comparisons. Exact P values are reported in the figure.

Having identified miR‐146a‐5p as a key candidate altered in ONFH, we next sought to characterize its functional relevance in BMSC fate and senescence. ONFH‐BMSCs display a senescence‐associated lineage skewing. Compared with BMSCs from DDH controls, ONFH‐BMSCs exhibited marked impairments in osteogenic differentiation capacity, as evidenced by reduced alkaline phosphatase (ALP) and Alizarin Red staining (Figure 2A,B). In parallel, Oil Red O staining revealed enhanced adipogenic differentiation, while SA‐β‐galactosidase (SA‐β‐gal) staining indicated elevated cellular senescence (Figure 2C,D). Consistent with these phenotypic shifts, RT‐qPCR showed downregulation of osteogenic genes (ALP, RUNX2, and OCN), upregulation of adipogenic genes (FABP4 and PPARG), and senescence markers (P16 and P21) in ONFH‐BMSCs (Figure 2E). These data indicated a pathological reprogramming of lineage commitment in ONFH‐BMSCs, favoring adipogenesis and cellular aging at the expense of osteogenesis.

Given the importance of extracellular vesicle (EV)‐mediated communication in regulating bone microenvironment homeostasis, we next isolated exosomes from the conditioned media of SLE‐ONFH and DDH‐BMSCs (Figure 2F). RT‐qPCR analysis confirmed that miR‐146a‐5p was significantly reduced in exosomes derived from ONFH‐BMSCs (Figure 2G). To evaluate their functional effects, exosomes were co‐cultured with healthy BMSCs. While DDH‐derived exosomes promoted osteogenic differentiation and reduced senescence in recipient cells, ONFH‐derived exosomes failed to exert such benefits (Figure 2H–K).

miR‐146a‐5p mediated the functional effects of BMSC exosomes. To specifically interrogate the role of miR‐146a‐5p, we silenced miR‐146a‐5p in DDH‐derived exosomes using siRNA, which abolished their regenerative effects. Conversely, engineering ONFH‐derived exosomes by supplementing miR‐146a‐5p mimics restored their ability to enhance osteogenesis and suppress senescence (Figure 2H–K). Gene expression analysis further confirmed that ALP, RUNX2, and OCN levels were rescued, while FABP4, PPARG, P16, and P21 were suppressed after treatment with miR‐146a‐5p‐enriched exosomes (Figure 2L).

These findings established that exosomes depletion of miR‐146a‐5p is a key driver of BMSCs aging and lineage imbalance in GC‐induced ONFH. Importantly, targeted restoration of miR‐146a‐5p within exosomes rescues the dysfunctional stem cell phenotype, suggesting a therapeutic avenue for regenerative intervention.

### 2.3 Engineered Exosome Delivery of miR‐146a‐5p Restored Stem Cell Function in ONFH‐BMSCs

**FIGURE 3:**
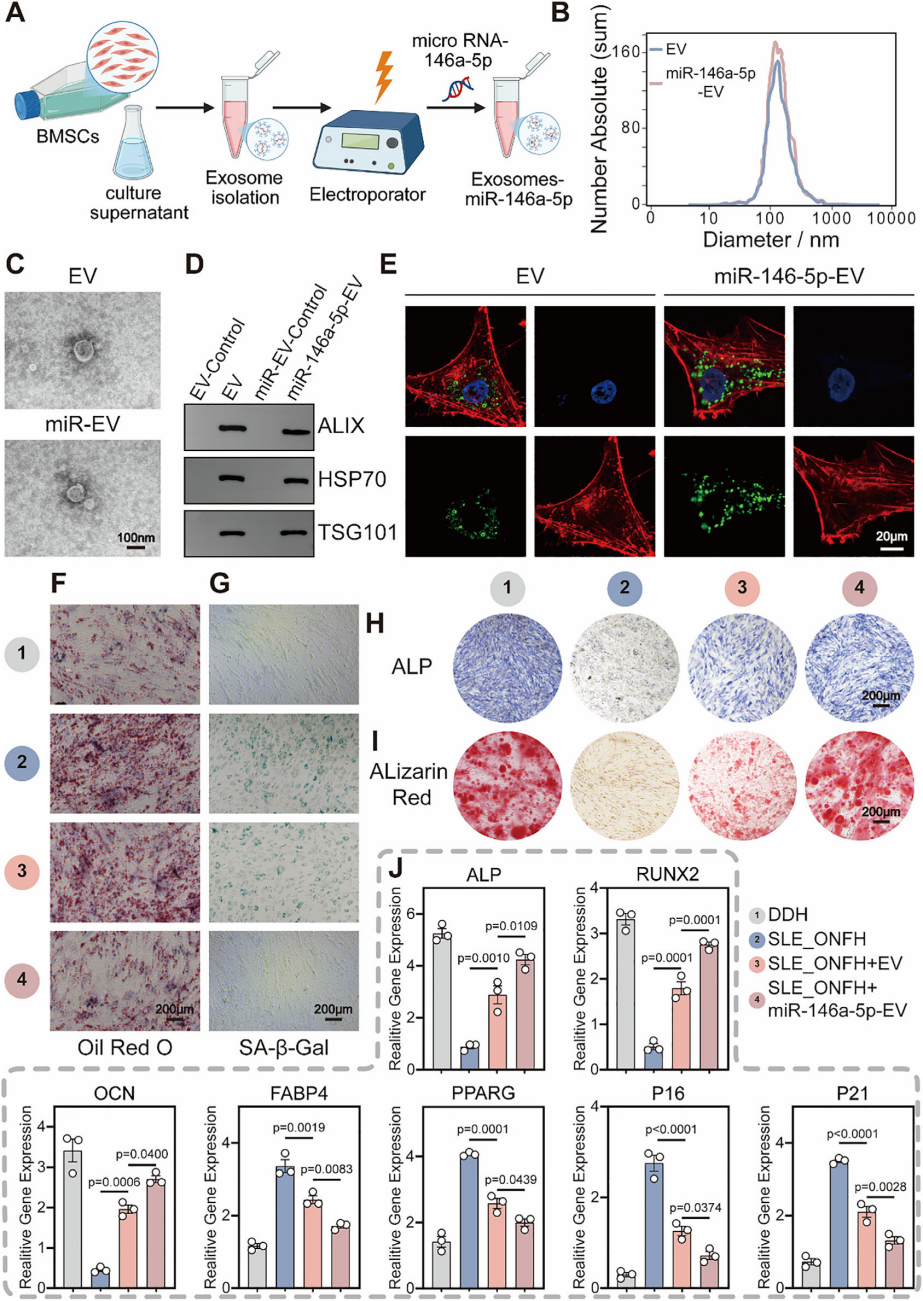
Engineered exosomal delivery of miR‐146a‐5p rescues stem cell dysfunction in ONFH‐BMSCs. (A) Schematic illustration of exosome engineering. BMSC‐derived exosomes were isolated and electroporated with synthetic miR‐146a‐5p mimics. (B) Nanoparticle tracking analysis showing comparable size distributions between engineered and control exosomes. (C) TEM images demonstrated preserved morphology after electroporation. TEM, transmission electron microscopy. (D) Western blot analysis confirmed expression of exosome markers (ALIX, HSP70 and TSG101) across groups. (E) Confocal microscopy images showed uptake of fluorescently labeled engineered exosomes by ONFH‐BMSCs after 24 h. (F and G) Representative images of Oil Red O staining (adipogenesis) and SA‐β‐gal staining (senescence) in ONFH‐BMSCs treated with indicated exosomes. (H and I) Representative images of ALP staining and Alizarin Red staining showing osteogenic differentiation outcomes after treatment. (J) RT‐qPCR analysis of osteogenic (ALP, RUNX2 and OCN), adipogenic (FABP4 and PPARG,) and senescence (P16 and P21) gene expression in treated BMSCs (n = 3). Data are presented as mean ± SEM. One‐way ANOVA with Tukey's post hoc test was used for multiple comparisons. Exact P values are reported in the figure.

Given the critical regulatory role of miR‐146a‐5p in BMSC homeostasis, we next developed an engineered exosome platform to deliver miR‐146a‐5p into ONFH‐BMSCs and evaluated its therapeutic efficacy. Exosomes were isolated from the culture supernatant of DDH BMSCs and loaded with synthetic miR‐146a‐5p mimics via electroporation, generating miR‐146a‐5p‐enriched exosomes (miR‐146a‐5p‐EVs) (Figure 3A). Nanoparticle tracking analysis revealed no significant change in size distribution between control EVs and miR‐146a‐5p‐EVs (Figure 3B), and transmission electron microscopy (TEM) confirmed that the vesicular morphology remained intact after electroporation (Figure 3C). Western blot further validated the expression of canonical exosome markers, including ALIX, HSP70, and TSG101 in both groups (Figure 3D). RT‐qPCR also showed successful loading of miR‐146a‐5p (Figure S2).

To verify functional delivery, BMSCs were incubated with fluorescently labeled exosomes. Confocal microscopy demonstrated efficient uptake of miR‐146a‐5p‐EVs (stained with green) into the cytoplasm within 24 h (Figure 3E), confirming successful internalization. Functionally, treatment of ONFH‐BMSCs with miR‐146a‐5p‐EVs resulted in a marked improvement in their regenerative properties. Oil Red O and SA‐β‐gal staining revealed that miR‐146a‐5p‐EVs significantly suppressed adipogenic differentiation and cellular senescence compared to untreated or control EV‐treated cells (Figure 3F, 3G). Correspondingly, ALP and Alizarin Red S staining showed enhanced osteogenic capacity (Figure 3H,I). At the molecular level, RT‐qPCR analysis demonstrated upregulation of osteogenic genes (ALP, RUNX2, and OCN), alongside downregulation of adipogenic (FABP4 and PPARG) and senescence‐associated (p16 and p21) markers (Figure 3J). Notably, these effects were significantly superior to those achieved by control EVs, confirming that miR‐146a‐5p loading was essential for therapeutic activity. To distinguish sequence‐specific effects of miR‐146a‐5p from potential effects of exosomal miRNA loading, exosomes carrying a non‐targeting miRNA (miR‐NC‐EVs) were included in osteogenic differentiation assays. miR‐NC‐EVs did not significantly enhance osteogenic outcomes compared with control EVs (Figure S3).

These results established that exosome delivery of miR‐146a‐5p effectively rejuvenates ONFH‐BMSCs by reversing GC‐induced senescence and restoring lineage balance. The engineered exosome platform offers a feasible, cell‐free approach to deliver functional RNA therapeutics for skeletal tissue regeneration.

### 2.4 Engineered Exosomes Enriched With miR‐146a‐5p Promote Angiogenic Activation of Endothelial Cells

Effective skeletal regeneration depends not only on osteogenesis but also on coordinated vascular remodeling to support tissue repair. Given the dual roles of miR‐146a‐5p‐enriched exosomes in BMSC rejuvenation, we next examined their impact on endothelial cell function, a key determinant of angiogenesis. Human umbilical vein endothelial cells (HUVECs) treated with MPS were incubated with control exosomes or miR‐146a‐5p‐enriched exosomes, followed by assessment of migratory and angiogenic behaviors.

**FIGURE 4:**
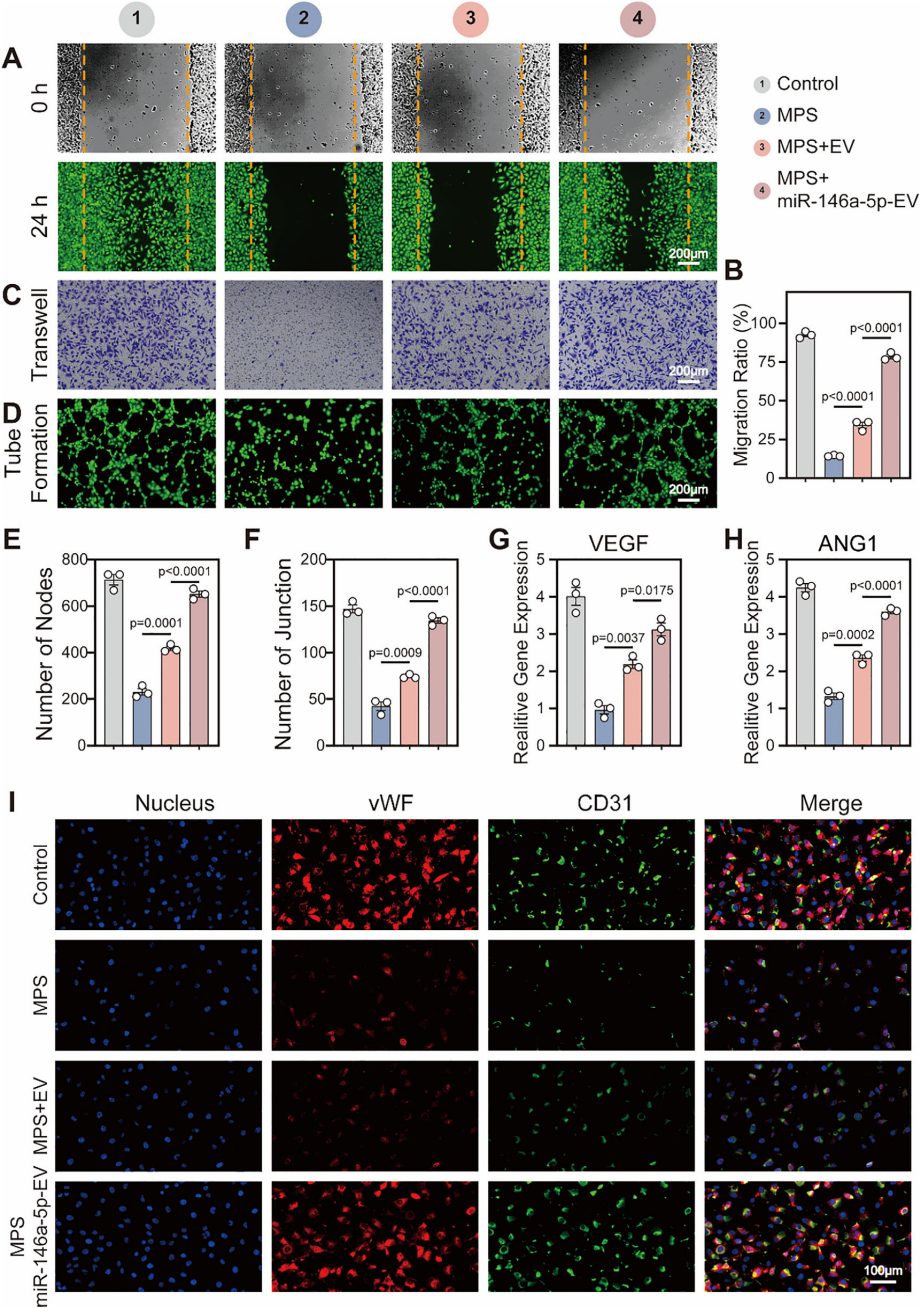
miR‐146a‐5p enriched exosomes enhanced endothelial migration and angiogenesis. (A) Representative images of scratch wound healing assays at 0 h and 24 h showing enhanced migration of HUVECs after treatment with miR‐146a‐5p enriched exosomes. HUVECs, human umbilical vein endothelial cells. (C) Representative images of transwell migration assays quantifying endothelial transmembrane migration. (D) Representative images of Matrigel tube formation assays showing vascular network formation following different treatments. (B, E and F) Quantification of tube formation parameters, including migration ratio (B), number of nodes (E) and number of junction (F) (n = 3). (G and H) RT‐qPCR analysis of angiogenesis‐related gene expression (VEGF and ANG1) in HUVECs after treatment (n = 3). (I) Representative immunofluorescence images showing vWF and CD31 expression and localization in treated endothelial cells. Data are presented as mean ± SEM. One‐way ANOVA with Tukey's post hoc test was used for multiple comparisons. Exact P values are reported in the figure.

In scratch wound healing assays, HUVECs exposed to miR‐146a‐5p‐EVs exhibited significantly accelerated wound closure compared to both untreated and control EV‐treated groups (Figure 4A,B), indicating enhanced migratory capacity. Similarly, Transwell migration assays demonstrated a higher number of migrating cells upon miR‐146a‐5p‐EV treatment (Figure 4C), further corroborating the pro‐migratory effect.

Moreover, Matrigel tube formation assays revealed robust promotion of capillary‐like structure formation following miR‐146a‐5p‐EV stimulation. Treated HUVECs exhibited increased numbers of nodes and junctions, alongside greater overall tube complexity and stability (Figure 4D–F). At the molecular level, RT‐qPCR analysis showed that key pro‐angiogenic genes, including VEGF and ANG1, were significantly upregulated in HUVECs treated with miR‐146a‐5p‐EVs (Figure 4G,H). Immunofluorescence staining confirmed enhanced expression and membrane localization of vascular endothelial markers, including angiogenic marker vWF and CD31 (Figure 4I), suggesting activation of endothelial programs critical for vessel formation.

These findings demonstrated that engineered miR‐146a‐5p‐enriched exosomes directly enhanced endothelial migratory and angiogenic potential. By simultaneously rejuvenating BMSCs and stimulating vascular remodeling, miR‐146a‐5p‐EVs orchestrated a comprehensive restoration of the osteogenic‐angiogenic microenvironment necessary for effective bone repair.

### 2.5 Intraosseous Delivery of Engineered miR‐146a‐5p Exosomes Restored Gait Function and Bone Architecture in a Rat Model of GC‐Induced ONFH

**FIGURE 5:**
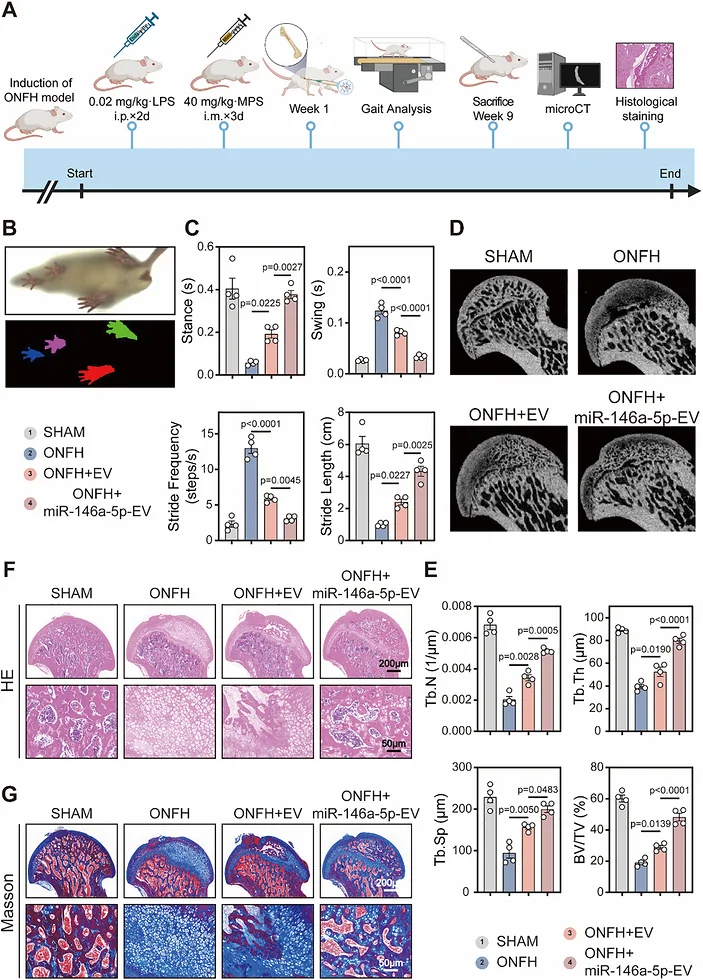
Intraosseous delivery of engineered miR‐146a‐5p exosomes restored gait function and bone architecture in a rat model of GC‐induced ONFH (A) Schematic of ONFH model induction and exosome treatment protocol. Rats received sequential LPS and MPS injections to induce ONFH, followed by intraosseous injection of engineered miR‐146a‐5p exosomes or controls. LPS, lipopolysaccharide; MPS, methylprednisolone. (B) Representative images of gait analysis by DigiGait imaging system. (C) Quantification of gait parameters, including stance time, swing time, stride frequency and stride length (n = 4). (D) Representative microCT images showing bone trabecular structure of the femoral head across groups (n = 4). (E) Quantification of trabecular bone parameters including trabecular number (Tb.N), trabecular thickness (Tb.Th), trabecular separation (Tb.Sp) and bone volume fraction (BV/TV) (n = 4). (F) Representative H&E staining of femoral heads showing trabecular integrity and marrow structure. (G) Representative Masson's trichrome staining demonstrating collagen deposition and tissue organization. Data are presented as mean ± SEM. One‐way ANOVA with Tukey's post hoc test was used for multiple comparisons. Exact P values are reported in the figure.

To validate the therapeutic potential of miR‐146a‐5p‐enriched exosomes in vivo, we established a rat model of GC‐induced ONFH by sequential administration of lipopolysaccharide (LPS) and MPS, mimicking clinical disease progression (Figure 5A). Following model induction, engineered exosomes or control exosomes were delivered via intraosseous injection directly into the femoral head. To further confirm the local delivery and intramedullary distribution of exosomes following intraosseous administration, fluorescence tracing was performed on isolated femora at day 1 and day 3 after injection of DIR‐labeled exosomes. Fluorescence signals were initially detected along the femoral shaft and subsequently redistributed toward the femoral neck and femoral head region, indicating localized retention within the target bone compartment (Figure S4). MiR‐146a‐5p exosomes ameliorated functional gait impairment. Behavioral gait analysis revealed that GC‐induced ONFH rats exhibit significant motor deficits, characterized by shortened stance time, prolonged swing time, increased stride frequency, and shortened stride length compared to sham controls (Figure 5B,C). Strikingly, treatment with miR‐146a‐5p‐enriched exosomes significantly improved all gait parameters, restoring locomotor function toward normal levels, whereas control exosomes showed only partial effects. These data suggested that exosomal miR‐146a‐5p delivery alleviates ONFH‐induced biomechanical dysfunction.

After euthanasia of the animals, micro‐computed tomography (microCT) imaging demonstrated severe trabecular disruption, cortical thinning, and subchondral collapse in untreated ONFH femoral heads. In contrast, engineered exosome treatment markedly preserved trabecular continuity and overall bone structure (Figure 5D). Quantitative analysis confirmed a significant increase in trabecular number (Tb.N) and trabecular thickness (Tb.Th), alongside reduced trabecular separation (Tb.Sp), culminating in improved bone volume fraction (BV/TV) (Figure 5E). Same as above, hematoxylin and eosin (H&E) staining revealed marked trabecular disruption accompanied by extensive osteocyte degeneration in ONFH rats, characterized by empty lacunae and prominent vacuole‐like changes within the bone marrow space. These features are consistent with glucocorticoid‐induced osteocyte injury, including nuclear condensation and cytoplasmic clearing. Notably, immunohistochemical staining of Perilipin 2 suggested the involvement of lipid‐associated changes in regions exhibiting these vacuole‐like structures (Figure S5). Treatment with miR‐146a‐5p exosomes markedly reduced necrotic areas, preserved trabecular architecture, and restored cellular density within the bone marrow compartment (Figure 5F). Masson's trichrome staining further demonstrated decreased fibrotic deposition and increased collagen organization, consistent with active tissue repair (Figure 5G). To further evaluate the in vivo safety of engineered exosome administration, hematological parameters, serum biochemical parameters, and histological analyses of major organs were assessed. No significant alterations in blood routine parameters or liver and kidney function markers, nor obvious histopathological abnormalities in major organs, were observed following treatment (Figure S6).

Collectively, these data demonstrated that local delivery of miR‐146a‐5p enriched exosomes robustly restores femoral head structure, alleviates motor dysfunction, and promotes tissue regeneration in a clinically relevant ONFH model. The observed improvements highlight the dual osteogenic and angiogenic regenerative capacity of exosome‐based therapies targeting stem cell dysfunction.

### 2.6 miR‐146a‐5p‐Enriched Exosomes Promoted Coordinated Osteogenesis and Angiogenesis In Vivo

**FIGURE 6:**
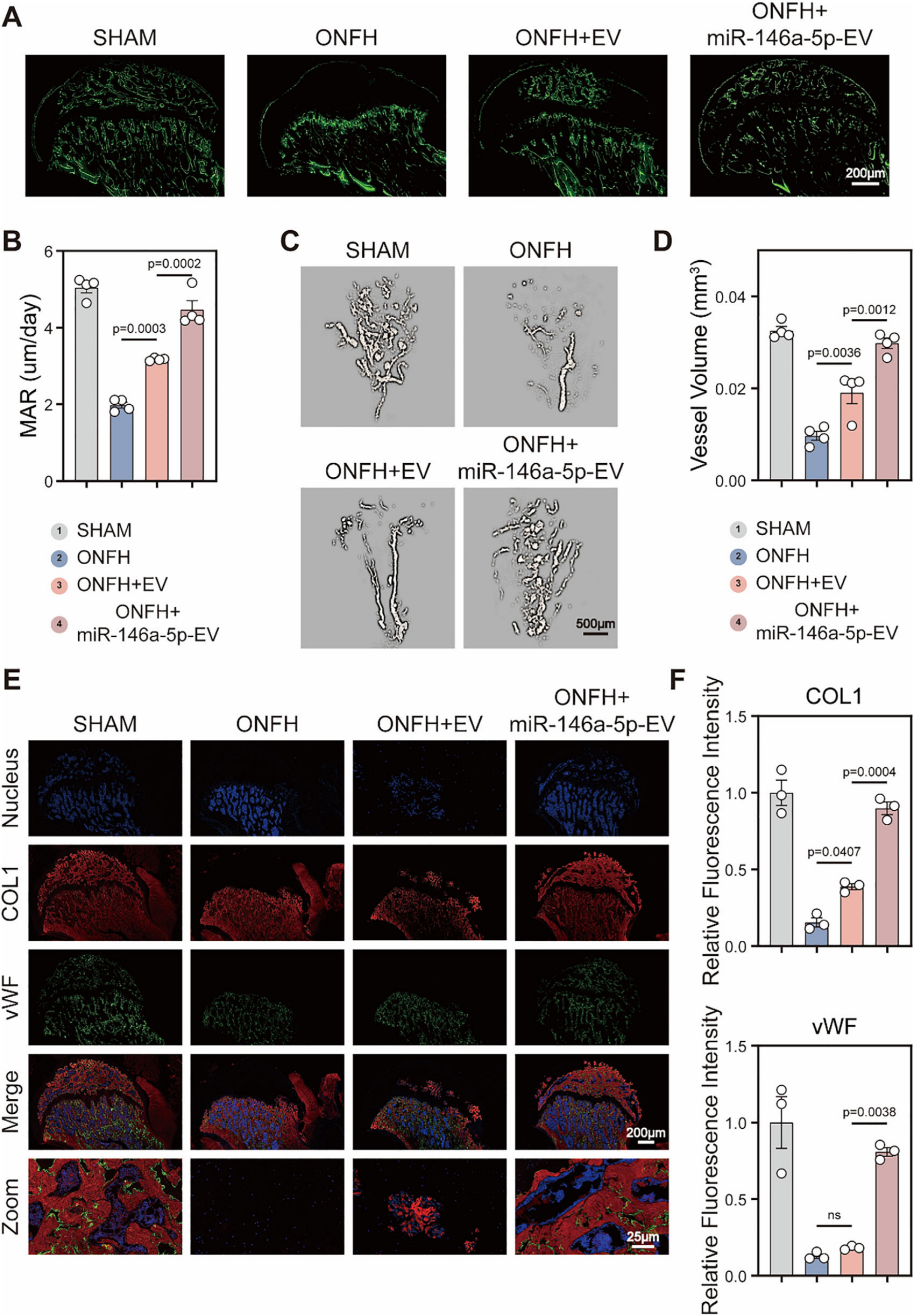
MiR‐146a‐5p enriched exosomes promoted coordinated osteogenesis and angiogenesis in vivo (A) Representative calcein double labeling images of femoral heads across treatment groups, visualizing mineralizing bone surfaces. (B) Quantification of mineral apposition rate based on calcein labeling (n = 4). (C) Representative Microfil perfusion images of intraosseous vasculature in femoral heads.(D) Quantification of vessel volume (n = 4). (E) Immunofluorescence staining for COL1 (red) and vWF (green) in femoral head sections. Nuclei were counterstained with DAPI (blue). Zoomed images highlight osteo‐angiogenic structures. (F) Quantification of relative fluorescence intensities for COL1 and vWF (n = 4). Data are presented as mean ± SEM. One‐way ANOVA with Tukey's post hoc test was used for multiple comparisons. Exact P values are reported in the figure; ns, not significant.

To further characterize the regenerative effects of miR‐146a‐5p‐enriched exosomes at the cellular level, we performed dynamic bone labeling, microvascular imaging, and immunofluorescence staining in the rat ONFH model. Dynamic bone formation was assessed by calcein double labeling. ONFH rats exhibited markedly reduced mineral apposition rate (MAR), reflecting impaired new bone formation. Treatment with engineered exosomes significantly enhanced MAR, restoring it to levels comparable with sham‐operated controls (Figure 6A,B). Microfil‐based vascular casting revealed that ONFH femoral heads displayed sparse and fragmented vascular networks. By contrast, the miR‐146a‐5p‐enriched exosome group showed robust restoration of intraosseous vessel volume and complexity (Figure 6C.D), suggesting effective vascular remodeling. Immunofluorescence staining further corroborated these findings. In ONFH rats, expression of osteogenic marker COL1 and vWF was severely diminished. Exosome treatment markedly upregulated COL1 and vWF expression, with clear co‐localization along newly formed trabeculae and vascular structures (Figure 6E,F). Quantitative analysis confirmed significant increases in both osteogenic and angiogenic signal intensities, indicating coordinated activation of bone and vascular niches essential for skeletal repair.

MiR‐146a‐5p‐enriched exosomes not only enhanced bone matrix deposition but also stimulated vascular regeneration, achieving synchronized osteo‐angiogenic repair of the necrotic femoral head microenvironment.

### 2.7 Single‐Cell Transcriptomics Reveals Lineage Rebalancing and Stem Cell Subset Remodeling by Engineered Exosomes In Vivo

**FIGURE 7:**
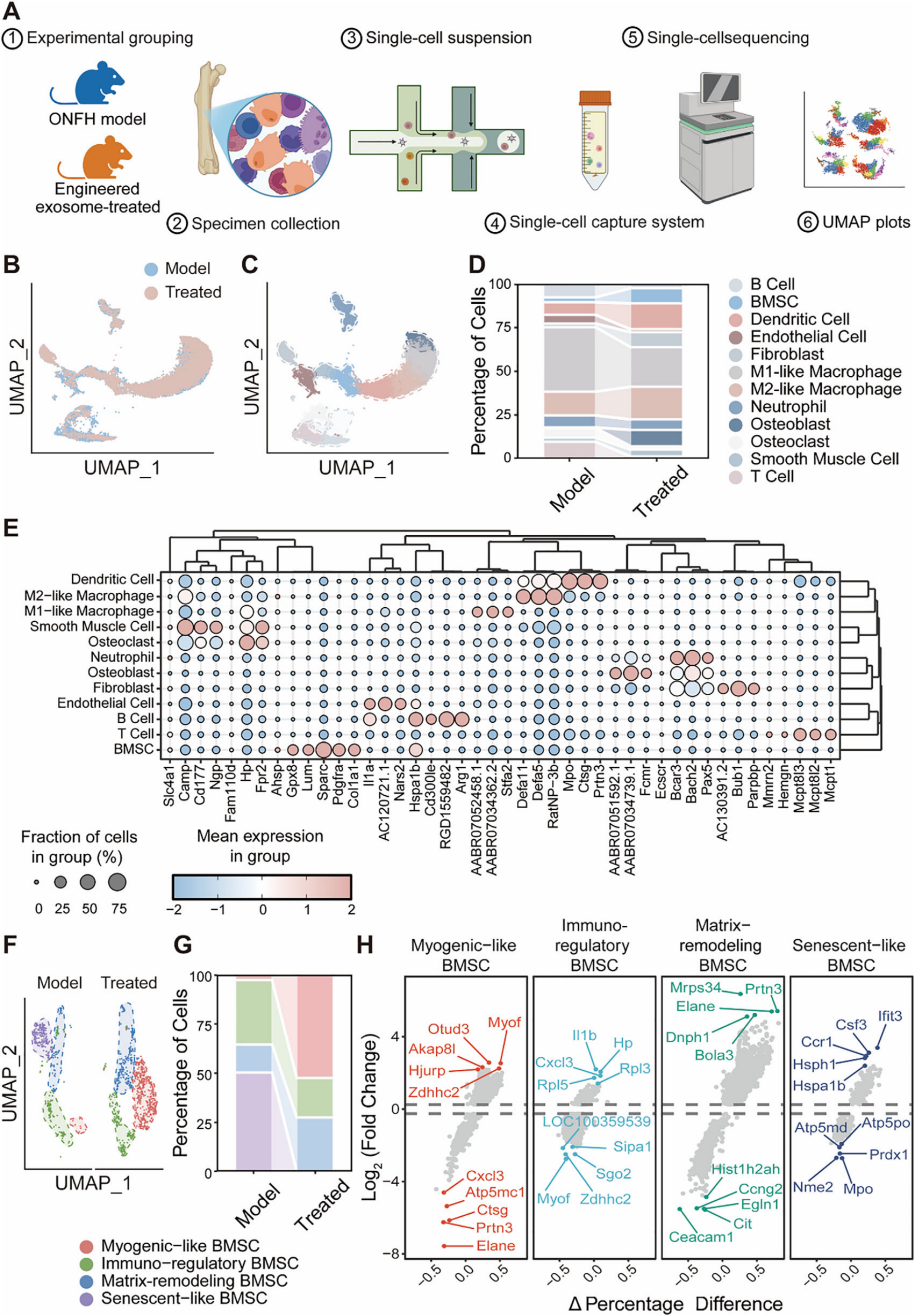
Single‐cell transcriptomics reveals lineage rebalancing and stem cell subset remodeling by engineered exosomes in vivo (A) Schematic of experimental workflow. ONFH rats were treated with engineered exosomes and femoral head tissues underwent single‐cell RNA sequencing. (B and C) UMAP plots showing global cell populations in ONFH model and exosome‐treated rats. (D) Cell composition shifts following exosome therapy, highlighting restoration of BMSC and osteoblast populations. (E) Heatmap of top differentially expressed genes across major cell types. (F) UMAP of refined BMSC clusters, identifying myogenic‐like, immunoregulatory, matrix‐remodeling and senescent‐like subsets. (G) Proportional changes in BMSC subpopulations between groups. (H) Volcano plots showing subset‐specific gene expression changes following exosome treatment, emphasizing expansion of osteogenic and suppression of senescent programs.

To elucidate the cellular basis underlying the regenerative effects of engineered miR‐146a‐5p‐enriched exosomes, we performed single‐cell RNA sequencing (scRNA‐seq) on femoral head tissues from GC‐induced ONFH model rats, comparing untreated (model) and exosome‐treated groups(treated) (Figure 7A). Unbiased clustering and UMAP projection revealed a diverse landscape composed of BMSCs, osteoblasts, osteoclasts, endothelial cells, fibroblasts, and multiple immune cell subsets (Figure 7B,C). Notably, ONFH samples exhibited a marked reduction in BMSCs and osteoblasts, accompanied by expansion of inflammatory cells, including M1‐like macrophages and neutrophils. Exosome treatment partially restored the stromal compartment, with increased representation of BMSCs and osteoblasts and a concomitant decrease in inflammatory subsets (Figure 7D), suggesting that engineered exosomes promoted rebalancing of the disrupted bone marrow niche.

To further characterize transcriptional remodeling across cell types, we analyzed hallmark gene expression signatures. Compared to untreated ONFH samples, exosome‐treated tissues displayed upregulation of osteogenic and angiogenic markers such as LUM, SPARC, and COL1a1, while expression of inflammatory mediators, including CD177 and NGP were significantly suppressed (Figure 7E). These changes implied that exosome therapy not only restores cellular composition but also reprograms functional gene expression toward a pro‐regenerative profile.

Focusing specifically on the BMSC compartment, we identified four major transcriptional subclusters, which are myogenic‐like BMSCs enriched for osteogenic programs, immunoregulatory BMSCs associated with immune modulation, matrix‐remodeling BMSCs producing extracellular matrix components, and senescent‐like BMSCs characterized by stress and aging signatures (Figure 7F). In ONFH rats, senescent‐like BMSCs predominated, while myogenic and matrix‐remodeling subsets were diminished. In contrast, exosome treatment significantly expanded the regenerative BMSC subsets while contracting the senescent‐like population (Figure 7G). Gene expression analysis within the BMSC subset revealed that exosome therapy enriched osteogenesis‐related genes (e.g., Myof) in the myogenic‐like cluster and extracellular matrix assembly genes (e.g., Prtn3, Elane) in the matrix‐remodeling cluster (Figure 7H). Notably, these transcriptional changes were observed as coordinated shifts in subset abundance and functional gene programs, rather than evidence of direct, cell‐intrinsic conversion between distinct BMSC states. The redistribution of BMSC subpopulations is more plausibly explained by an improved marrow microenvironment following miR‐146a‐5p exosome delivery, including attenuation of inflammatory stress and restoration of mitochondrial homeostasis, conditions known to favor osteogenic competence while suppressing senescence‐associated programs. In this context, engineered miR‐146a‐5p exosomes appear to bias BMSC functional states toward regenerative trajectories without enforcing deterministic lineage reprogramming at the single‐cell level.

Collectively, these single‐cell data demonstrated that exosome therapy restores bone marrow stromal homeostasis in vivo by both rebalancing major cell populations and promoting lineage‐specific rejuvenation within the BMSC compartment, thereby laying the cellular foundation for functional bone repair in GC‐induced ONFH.

### 2.8 Engineered miR‐146a‐5p Exosomes Globally Reprogram the Transcriptome of ONFH‐BMSCs Toward a Restored Functional State

**FIGURE 8:**
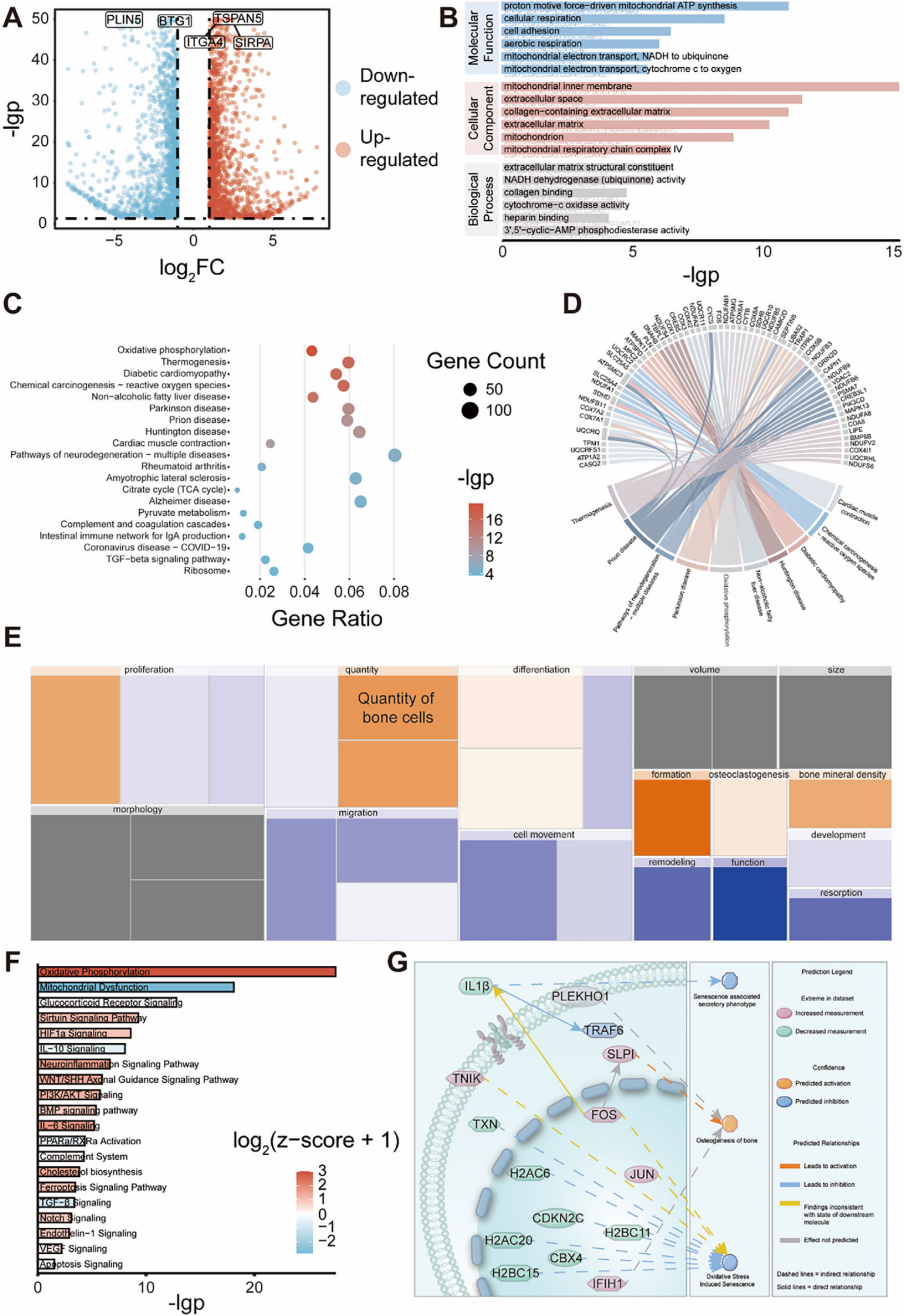
Engineered miR‐146a‐5p exosomes globally reprogram the transcriptome of ONFH‐BMSCs toward a regenerative state. (A) Volcano plot showing differentially expressed genes between ONFH‐BMSCs treated with engineered miR‐146a‐5p exosomes versus untreated ONFH‐BMSCs. Red and blue dots represent significantly upregulated and downregulated genes, respectively. (B) Gene Ontology enrichment analysis of upregulated and downregulated genes based on biological process, cellular component and molecular function categories. (C) KEGG pathway enrichment analysis highlighting major biological pathways altered upon exosome treatment, including upregulation of oxidative phosphorylation and ribosome biogenesis. (D) Circular chord diagram depicting relationships between key differentially expressed genes and enriched KEGG pathways. (E) Functional annotation clustering summarizing major biological changes after treatment, including increased bone cell proliferation, differentiation and migration. (F) IPA canonical pathway enrichment identifying activation of mitochondrial function and inhibition of senescence‐ and stress‐related signaling. IPA, ingenuity pathway analysis (G) IPA upstream regulator analysis predicting inhibition of pro‐senescent mediators (TRAF6 and IL‐1β) and activation of bone regenerative transcription factors (FOS and JUN) following exosome treatment.

To gain a comprehensive understanding of how engineered miR‐146a‐5p‐enriched exosomes modulate the molecular landscape of dysfunctional ONFH‐BMSCs, we performed bulk RNA sequencing on BMSCs isolated from untreated and exosome‐treated ONFH rat femoral heads. Differential expression analysis revealed profound transcriptomic remodeling after exosome intervention, with a large number of genes significantly upregulated or downregulated, suggesting a global reprogramming effect. Notably, genes related to osteogenesis and extracellular matrix organization, such as ITGA4, TSPAN5, and SIRPA, were markedly induced, whereas transcripts associated with senescence, oxidative stress, and adipogenic drift, like PLIN5, were significantly suppressed in the exosome‐treated group (Figure 8A).

Gene ontology enrichment analysis showed that upregulated genes in the exosome‐treated group were predominantly involved in biological processes critical for tissue regeneration, including oxidative phosphorylation, extracellular matrix organization, skeletal system development, and mitochondrial function (Figure 8B). Kyoto encyclopedia of genes and genomes (KEGG) pathway enrichment further supported activation of mitochondrial metabolic pathways and ribosomal biogenesis, indicating a shift toward enhanced bioenergetic capacity and anabolic function in BMSCs following exosome treatment (Figure 8C). Visualization of differentially expressed genes in a circular chord diagram revealed coordinated regulation of key metabolic and regenerative modules, emphasizing the broad and interconnected nature of transcriptomic reprogramming (Figure 8D).

Functional annotation based on Ingenuity Pathway Analysis (IPA) highlighted that exosome‐treated BMSCs exhibited enhanced bone cell proliferation, differentiation, migration, and extracellular matrix deposition (Figure 8E). Canonical pathway analysis pinpointed the activation of oxidative phosphorylation and inhibition of mitochondrial dysfunction as major molecular shifts underlying the phenotypic restoration (Figure 8F). Finally, upstream regulator analysis suggested that the therapeutic effect of miR‐146a‐5p‐enriched exosomes is mediated through the inhibition of pro‐senescent and pro‐inflammatory mediators, such as TRAF6 and IL‐1β, and activation of bone anabolic regulators, including FOS and JUN (Figure 8G), providing mechanistic insights into how miR‐146a‐5p orchestrates tissue rejuvenation at the transcriptional level.

Collectively, these findings revealed that engineered miR‐146a‐5p exosomes impose a coordinated, multi‐layered reprogramming of ONFH‐BMSC transcriptomes, characterized by the activation of osteogenic, metabolic, and anti‐senescent gene networks, thereby establishing a molecular foundation conducive to effective stem cell recovery and bone tissue regeneration.

### 2.9 Engineered miR‐146a‐5p Exosomes Restored Mitochondrial Homeostasis by Suppressing the TRAF6–NF‐κB Axis and Oxidative Stress

**FIGURE 9:**
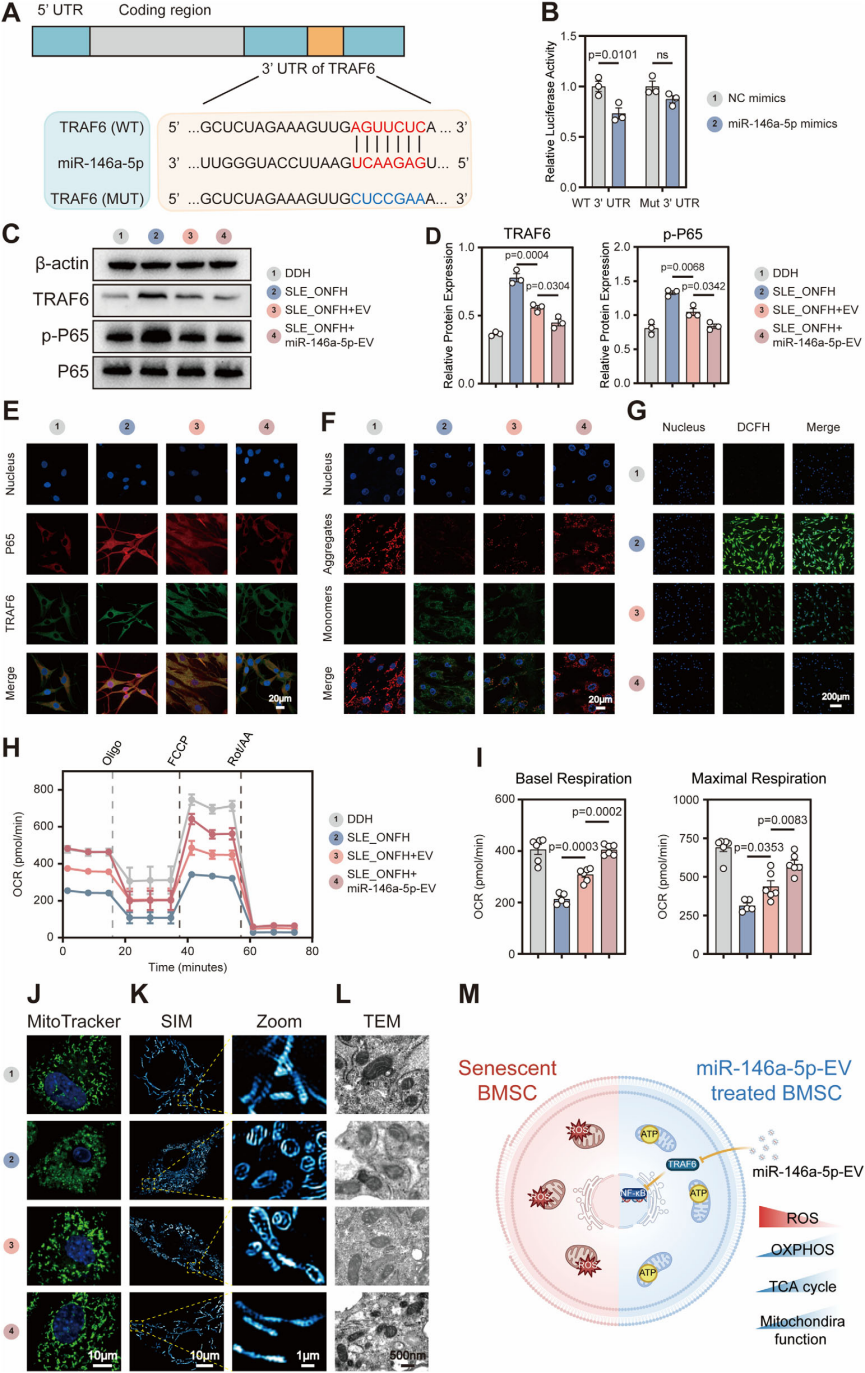
Engineered miR‐146a‐5p exosomes restored mitochondrial homeostasis by suppressing the TRAF6‐NF‐κB axis and oxidative stress (A) Schematic of luciferase reporter constructs containing the WT or Mut 3’ UTR of TRAF6 targeted by miR‐146a‐5p. WT, wild‐type; Mut, mutant. (B) Luciferase assay showing miR‐146a‐5p mediated repression of WT but not Mut TRAF6 3’ UTR (n = 3). (C and D) Western blot and quantification of TRAF6 and p‐p65 levels in BMSCs from indicated groups. p‐p65, phosphorylated p65 (n = 3). (E) Immunofluorescence staining of p65 (red) and TRAF6 (green) showing reduced nuclear translocation and cytoplasmic aggregation upon exosome treatment. (F) JC‐1 staining demonstrating restoration of mitochondrial membrane potential after exosome treatment. Red aggregates represent polarized mitochondria and green monomers indicate depolarization. (G) DCFH‐DA staining showing reduction of intracellular ROS levels with engineered exosome therapy. (H and I) Seahorse XF analysis of basal and maximal oxygen consumption rates, indicating mitochondrial respiratory function recovery (n = 6). (J) MitoTracker staining validating mitochondrial network recovery. (K and L) Structured illumination microscopy imaging and TEM analysis showing restored mitochondrial morphology with elongated, intact cristae after exosome treatment. (M) Schematic model illustrating miR‐146a‐5p mediated inhibition of the TRAF6‐NF‐κB‐ROS axis, promoting mitochondrial rejuvenation and stem cell survival. Data are presented as mean ± SEM. One‐way ANOVA with Tukey's post hoc test was used for multiple comparisons. Exact P values are reported in the figure; ns, not significant.

To delineate the molecular mechanism by which engineered miR‐146a‐5p exosomes restore mitochondrial integrity in ONFH‐BMSCs, we used multiple databases (miRWalk, Targeted Scan, mirDIP, and miRDB) for prediction, and the results showed that TRAF6 was included (Figure S7). We then confirmed direct targeting of TRAF6 by miR‐146a‐5p through a luciferase reporter assay, which showed that miR‐146a‐5p significantly suppressed the activity of the TRAF6 3’ UTR reporter but not the mutant construct (Figure 9A,B). Western blot analysis further demonstrated elevated TRAF6 and phosphorylated p65 (p‐p65) expression in ONFH‐BMSCs, consistent with activation of the NF‐κB inflammatory axis. Treatment with engineered miR‐146a‐5p exosomes markedly reduced both TRAF6 and p‐p65 levels (Figure 9C,D). Immunofluorescence staining revealed nuclear translocation of p65 and increased cytoplasmic TRAF6 aggregation in ONFH‐BMSCs, both of which were reversed by exosome treatment (Figure 9E). To further examine whether these effects are functionally dependent on TRAF6, rescue experiments were performed in ONFH‐BMSCs. MiR‐146a‐5p overexpression improved mitochondrial membrane potential and promoted osteogenic gene expression, whereas co‐expression of TRAF6 partially attenuated these protective effects. In addition, TRAF6 knockdown alone exerted beneficial effects, although to a lesser extent than miR‐146a‐5p restoration (Figure S8). These findings indicate that TRAF6 is a key mediator of miR‐146a‐5p–dependent regulation of mitochondrial homeostasis and BMSC fate. At the same time, to further confirm functional NF‐κB activation, we measured the mRNA levels of NF‐κB downstream target genes, including IL‐1β, TNF‐α, and IL‐6. As shown in Figure S9, these genes were significantly upregulated in ONFH‐BMSCs and markedly suppressed following engineered miR‐146a‐5p exosome treatment, supporting effective inhibition of NF‐κB signaling. These findings indicated that miR‐146a‐5p exosomal delivery effectively inhibits NF‐κB activation in pathological BMSCs.

Given the known role of NF‐κB in driving oxidative stress, we next assessed mitochondrial ROS levels and membrane potential. JC‐1 staining showed that ONFH‐BMSCs exhibited a higher proportion of green monomers, indicating mitochondrial depolarization and dysfunction. Engineered exosome treatment restored red JC‐1 aggregates, reflecting recovered mitochondrial membrane potential (Figure 9F). Concurrently, ROS levels detected by DCFH‐DA staining were markedly elevated in ONFH‐BMSCs and suppressed following exosome exposure (Figure 9G). To evaluate mitochondrial respiratory function, we performed Seahorse XF analysis. ONFH‐BMSCs displayed significant impairment in both basal and maximal oxygen consumption rates (OCR), which were significantly rescued after miR‐146a‐5p exosome administration (Figure 9H,I), indicating restored oxidative phosphorylation capacity. At the same time, ATP levels have also been rescued (Figure S10). Confocal microscopy, structured illumination microscopy (SIM), and TEM imaging, in turn, from different scales revealed that ONFH‐BMSCs exhibited swollen, fragmented mitochondria with disrupted cristae, while exosome‐treated cells showed elongated, interconnected mitochondrial networks with intact ultrastructure (Figure 9J‐L).

A working schematic summarizes the above findings. Engineered miR‐146a‐5p exosomes targeted TRAF6, inhibited NF‐κB activation, reduced ROS accumulation, and restored mitochondrial quality, thereby breaking the vicious cycle of oxidative damage and senescence in ONFH‐BMSCs (Figure 9M). These results collectively established that miR‐146a‐5p‐mediated suppression of the TRAF6–NF‐κB–ROS axis represents a central mechanism underlying mitochondrial rejuvenation and stem cell functional recovery.

### 2.10 Engineered miR‐146a‐5p Exosomes Activated Autophagy and Mitophagy to Restore Mitochondrial Quality Control in ONFH‐BMSCs

**FIGURE 10:**
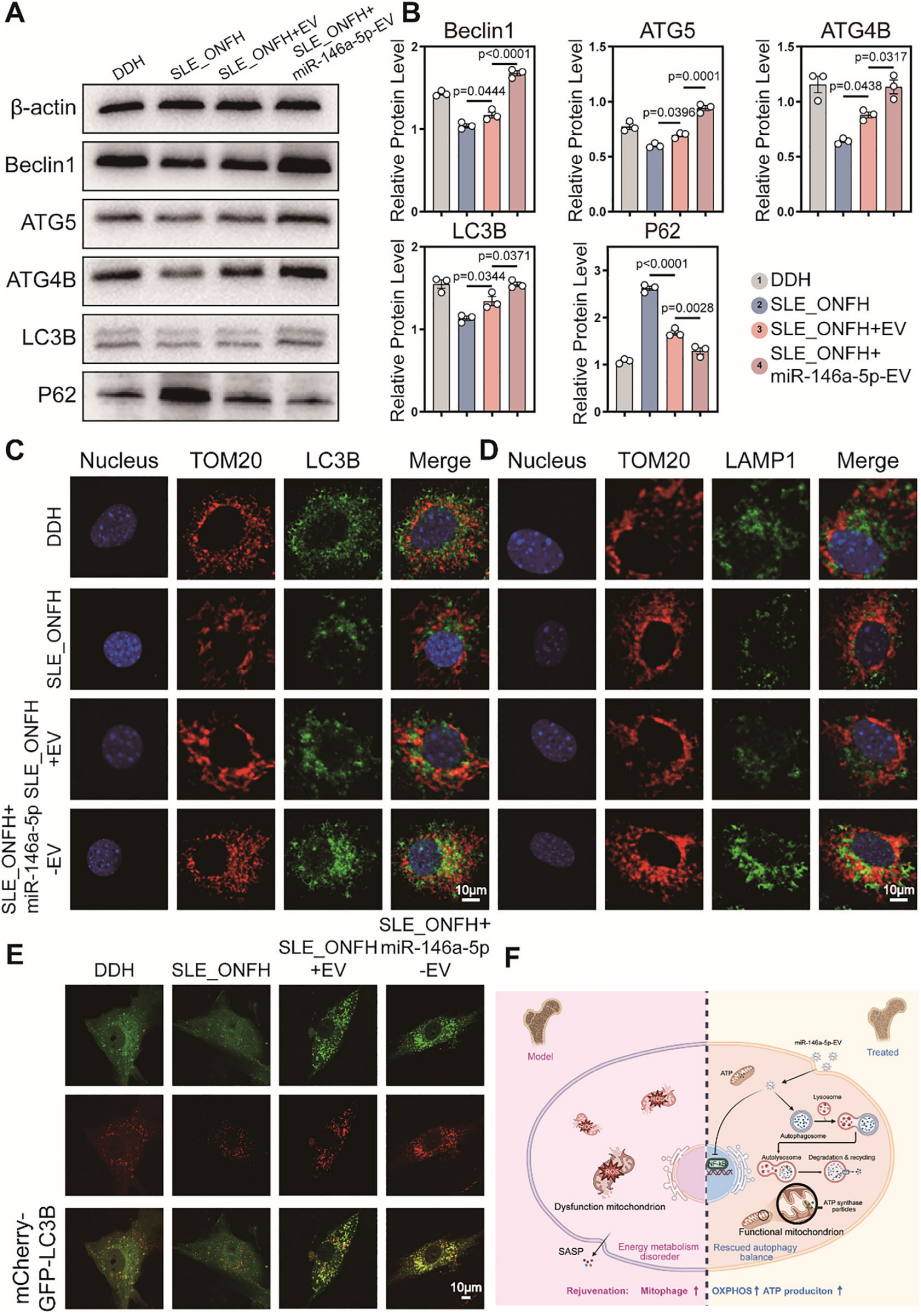
Engineered miR‐146a‐5p exosomes activated autophagy and mitophagy to restore mitochondrial quality control in ONFH‐BMSCs (A, B) Western blot and quantitative analysis of autophagy‐related proteins Beclin1, ATG5, ATG4B, LC3B and p62 in BMSCs from the indicated groups (n = 3). (C) Immunofluorescence staining of TOM20 (red) and LC3B (green) showing enhanced autophagosome formation and mitochondrial colocalization after exosome treatment. (D) Immunofluorescence staining of TOM20 (red) and LAMP1 (green) demonstrating increased mitochondria‐lysosome colocalization following exosome therapy. (E) Representative images of mCherry‐GFP‐LC3B reporter assay showing restoration of autophagic flux (red puncta) in ONFH‐BMSCs treated with engineered miR‐146a‐5p exosomes. (F) Schematic illustration of the proposed mechanism by which miR‐146a‐5p enriched exosomes suppress oxidative stress, restore mitochondrial function and reactivate autophagy/mitophagy to rejuvenate BMSC function under GC‐induced stress. Data are presented as mean ± SEM. One‐way ANOVA with Tukey's post hoc test was used for multiple comparisons. Exact P values are reported in the figure.

To further elucidate the downstream mechanisms by which engineered miR‐146a‐5p exosomes maintain mitochondrial homeostasis, we examined their impact on autophagy and mitophagy in ONFH‐BMSCs. Western blot revealed that ONFH‐BMSCs displayed a significant reduction in key autophagy‐related proteins, including Beclin1, ATG5, ATG4B, and the LC3B‐II/LC3B‐I ratio, along with elevated p62 accumulation, indicating impaired autophagic flux (Figure 10A,B). Treatment with engineered miR‐146a‐5p exosomes restored the expression of Beclin1, ATG5, and ATG4B, increased LC3‐II levels, and reduced p62 deposition, suggesting robust reactivation of the autophagy machinery.

Immunofluorescence analysis further supported these observations. Staining for TOM20 (a mitochondrial marker) and LC3B demonstrated sparse, weak LC3B puncta in ONFH‐BMSCs, whereas exosome treatment markedly increased LC3B‐positive autophagosomes, many of which colocalized with mitochondria, indicative of active mitophagy (Figure 10C). Similarly, co‐staining of TOM20 and LAMP1 showed enhanced lysosomal association with mitochondria following exosome therapy, reflecting successful autophagosome‐lysosome fusion (Figure 10D). To visualize autophagic dynamics more precisely, we employed mCherry‐GFP‐tagged LC3B reporter constructs. ONFH‐BMSCs showed few red‐only puncta, indicating defective autolysosome formation. In contrast, engineered exosome treatment increased the proportion of red puncta, confirming restoration of late‐stage autophagic flux (Figure 10E). In order to determine whether miR‐146a‐5p‐mediated mitochondrial protection is associated with mitophagy, ONFH‐BMSCs were treated with the mitophagy inhibitor Mdivi‐1. Pharmacological inhibition of mitophagy markedly attenuated the miR‐146a‐5p induced improvements in mitochondrial morphology, mitochondrial membrane potential, and intracellular ROS levels. Consistently, p62 protein levels were reduced in miR‐146a‐5p treated cells but accumulated following Mdivi‐1 treatment. These findings suggest that mitophagy contributes to the mitochondrial protective effects of miR‐146a‐5p (Figure S11).

Together, these results suggested that beyond reducing oxidative stress and stabilizing mitochondrial membranes, engineered miR‐146a‐5p exosomes actively promote the clearance of damaged mitochondria via autophagy and mitophagy. The mechanistic schematic summarized that miR‐146a‐5p mediated suppression of the TRAF6‐NF‐κB‐ROS axis, coupled with reactivation of mitochondrial turnover, collaboratively restores metabolic homeostasis, prevents senescence, and enhances regenerative potential in ONFH‐BMSCs (Figure 10F).

## 3 Discussion

GC‐induced ONFH is a debilitating condition characterized by bone necrosis in the femoral head following long‐term use of glucocorticoids such as MPS, dexamethasone, and hydrocortisone [39]. GCs are critical therapeutics for inflammatory and autoimmune diseases, yet their prolonged use is severely limited by adverse effects, particularly ONFH. Epidemiological data have revealed that using GC is one of the leading causes of non‐traumatic ONFH, representing a major clinical burden (accounting for approximately 51% of ONFH cases in Japan and 24.1% in China) [40, 41]. The pathogenesis of GC‐induced ONFH is multifactorial and complex. Classical theories suggested it involves coagulation disorders, apoptosis of osteoblasts as well as osteocytes, lipid metabolism dysfunction, and microvascular occlusion [42, 43]. Recent data, however, has highlighted the role of inflammation and stress signaling pathways. Despite well‐documented epidemiology, the molecular mechanisms of GC‐induced ONFH remain incompletely understood. Emerging evidence points to a combined contribution of inflammatory dysregulation, oxidative stress, mitochondrial dysfunction, and impaired autophagy pathways in GC‐induced skeletal damage [11, 44, 45, 46].

Specifically, we found that GC exposure induces mitochondrial dysfunction in BMSCs, consistent with recent reports identifying mitochondrial injury as a hallmark of ONFH [47, 48]. Healthy mitochondria are essential for the survival of osteoblasts, osteocytes, and BMSCs, as they generate ATP via oxidative phosphorylation, regulate calcium and redox homeostasis [49, 50]. In our study, GC treatment led to mitochondrial membrane potential loss and increased ROS, whereas miR‐146a‐5p treatment reversed these effects, stabilizing mitochondrial function and energy production. This aligns with the concept that targeting mitochondria through Parkin‐mediated mitophagy can ameliorate GC‐induced ONFH. To elucidate the mechanisms underlying GC‐induced ONFH, we performed multi‐omics analysis on BMSCs from SLE patients who developed ONFH after GC therapy. Integrated mRNA, miRNA, and lncRNA transcriptomics identified miR‐146a‐5p as one of the most significantly downregulated non‐coding RNAs in ONFH‐derived BMSCs. CeRNA network analysis revealed that miR‐146a‐5p acts as a central regulatory hub between inflammation and osteogenesis, linking critical lncRNAs (e.g., XIST, MALAT1) to downstream targets such as PTGFRN and ZNRF2. The downregulation of miR‐146a‐5p was validated in independent cohorts and detected in the serum of ONFH patients but not in GC‐treated SLE patients without ONFH or in those with developmental DDH, suggesting both mechanistic relevance and biomarker potential. The consistent downregulation in ONFH bone marrow and circulation, but not in non‐ONFH controls, highlights miR‐146a‐5p as a necrosis‐associated and disease‐relevant circulating biomarker.

Meanwhile, we observed that miR‐146a‐5p significantly attenuated inflammatory signaling in GC‐treated cells. Previous studies have identified miR‐146a‐5p as a negative regulator of innate inflammation, for instance, by targeting adaptor proteins IRAK1 or TRAF6 to suppress NF‐κB activation [33]. Consistently, delivery of miR‐146a‐5p reduced NF‐κB signaling and decreased proinflammatory cytokines such as IL‐1β and TNF‐α. We also noted suppressed activation of JAK/STAT3. Mechanistically, miR‐146a‐5p targeted key upstream activators of the NF‐κB pathway (e.g., IRAK1, TRAF6), inhibiting p65 nuclear translocation and proinflammatory cytokine release, thereby disrupting the pathological positive feedback loop among NF‐κB activation, ROS accumulation, and mitochondrial injury. Additionally, miR‐146a‐5p suppressed M1 macrophage polarization via the IRAK1/NF‐κB axis [51, 52]. These combined effects help break the vicious cycle of GC‐induced inflammation and cellular stress.

Autophagy represents another pivotal factor in the pathophysiology of GC‐ONFH, and our data showed that miR‐146a‐5p modulates this process. As a cellular housekeeping mechanism, autophagy removes damaged organelles and proteins, particularly under stress. In osteocytes and BMSCs, moderate autophagy supports survival, whereas excessive autophagic flux could cause cell death or dysfunction [53, 54]. Under GC treatment, we observed dysregulation of autophagic markers (abnormal LC3‐II and p62 levels), indicating autophagy imbalance. Remarkably, miR‐146a‐5p partially restored autophagy, normalizing LC3‐II levels and p62 degradation, which suggests a return to protective autophagy. The interplay among inflammation, oxidative stress, and autophagy is essential for BMSC homeostasis. Chronic GC exposure elevates intracellular ROS, overwhelms the autophagic system, and impairs the clearance of damaged mitochondria. TRAF6‐mediated K63‐linked ubiquitination of Beclin1 connects proinflammatory signaling to autophagy activation. MiR‐146a‐5p restored autophagy and mitophagy flux in GC‐stressed BMSCs by suppressing the TRAF6‐NF‐κB pathway. In addition to relieving oxidative stress, miR‐146a‐5p‐enriched EVs reactivated autophagy and mitophagy, restored Beclin1‐LC3 signaling, and enhanced autophagosome‐lysosome fusion, which is crucial for mitochondrial quality control under metabolic stress. These findings are consistent with prior reports that EV‐delivered autophagy modulators can rescue GC‐damaged cells. For example, BMSC‐derived EVs have been shown to activate autophagy in GC‐treated BMECs while inhibiting the PI3K‐Akt‐mTOR pathway, thereby preventing apoptosis [55]. By preventing autophagic dysfunction, miR‐146a‐5p helps protect against autophagic cell death. It should be noted that miR‐146a‐5p is unlikely to function through a single linear pathway in GC‐induced ONFH. Bioinformatic analyses indicate that miR‐146a‐5p may potentially interact with multiple mRNAs and lncRNAs involved in inflammation, mitochondrial stress, and cellular homeostasis, highlighting the complexity of its regulatory network (Tables S2 and S3). In the present study, we focused on the TRAF6–NF‐κB axis as a representative and mechanistically supported pathway relevant to inflammatory stress and mitochondrial dysfunction. Other regulatory routes may operate in parallel and warrant further investigation in future studies.

At the heart of these protective effects lies EV‐mediated intercellular communication. EVs, including exosomes, are known to shuttle proteins, RNAs, and lipids between cells in the bone microenvironment. In our study, EVs enriched in miR‐146a‐5p, possibly along with other cargos, were isolated and delivered to GC‐exposed osteoblasts, BMSCs, and endothelial cells, reprogramming them toward a reparative state. Functionally, miR‐146a‐5p‐deficient exosomes (derived from ONFH‐BMSCs) promoted BMSC senescence and adipogenic skewing, impairing osteogenic capacity. Restoration of miR‐146a‐5p reversed these phenotypes, confirming its causal role in stem cell dysfunction. Of particular note, the absence of miR‐146a‐5p in BMSC‐derived exosomes may itself constitute a pathological mechanism. Given that EVs serve as key communication vehicles in the bone marrow niche, impaired transport of miR‐146a‐5p via EVs likely exacerbates stem cell dysfunction in a non‐cell‐autonomous manner. ONFH‐derived EVs not only lacked miR‐146a‐5p but also failed to support osteogenesis or suppress senescence. By transferring miR‐146a‐5p, EVs directly inhibited inflammatory pathways and potentially targeted autophagy‐related genes to modulate autophagy. The ultimate result was reduced inflammatory cell infiltration and improved vascular integrity, in line with known effects of miR‐146a‐5p in reducing endothelial inflammation and permeability. Furthermore, EVs may carry other osteogenic or angiogenic regulators, further amplifying the repair signal. Overall, our findings highlight EVs as multifunctional delivery platforms orchestrating multiple protective pathways in GC‐affected bone tissue. We found that engineered exosomes enriched in miR‐146a‐5p effectively reversed GC‐induced BMSC dysfunction through coordinated regulation of inflammation, oxidative stress, and autophagy.

A crucial outcome of our intervention was the restoration of osteo‐angiogenic coupling. Bone regeneration relies heavily on the co‐growth of osteoblasts and blood vessels [55, 56]. Specialized “H‐type” capillaries in bone secrete angiocrine factors that sustain osteoprogenitors, while osteoblasts in turn secrete VEGF and PDGF to recruit vessels [56]. GC disrupts this interplay by injuring endothelial cells and suppressing VEGF signaling. GC also impairs vascular integrity in the femoral head. Recent studies identified lncRNA FAR591 as a key mediator of GC‐induced apoptosis in bone microvascular endothelial cells (BMECs). FAR591 was markedly upregulated under GC stimulation and formed RNA‐DNA triplex structures at the Fos promoter, enhancing its transcriptional activity. Fos then activated the pro‐apoptotic Bim‐PUMA axis, inducing mitochondria‐mediated apoptosis. Knockdown of FAR591 protected BMECs from apoptosis, preserved vascular structure, and alleviated ONFH, whereas overexpression exacerbated vascular injury. The FAR591‐Fos axis links GC exposure to femoral head vascular degradation [57].

In our model, GC inhibited endothelial cell migration, tube formation, and osteoblast differentiation. Strikingly, miR‐146a‐5p‐EVs reversed these defects, and we observed simultaneous increases in VEGF secretion, endothelial sprouting, and osteogenic markers. Beyond supporting osteogenesis, miR‐146a‐5p‐EVs directly enhanced endothelial functions, including migration, tube formation, and pro‐angiogenic gene expression (e.g., VEGF, vWF). These dual effects position miR‐146a‐5p as a key molecular coordinator of osteo‐angiogenic coupling. By mitigating GC‐driven inflammation and apoptosis, our treatment restored a regenerative bone microenvironment where osteogenesis and angiogenesis proceed in harmony. MiRNAs also play essential roles in bone‐immune interactions. Specific mesenchymal stem cell (MSC) subpopulations can modulate macrophage polarization and osteoclastogenesis, creating an immune microenvironment favorable for bone regeneration. MiR‐146a‐5p‐EVs may enhance local regenerative potential by reshaping the immune niche through alleviating chronic inflammation and oxidative stress.

In addition to intrinsic molecular mechanisms and direct effects of exogenous GC, host systemic factors, particularly the gut microbiome via the “gut‐bone axis”, have been implicated in ONFH. Intriguingly, probiotic‐derived EVs (e.g., from Lactobacillus animalis) carry bioactive factors that promote osteogenesis and angiogenesis [58]. In mouse models, probiotic supplementation or co‐housing restored microbiota‐derived EVs and ameliorated GC‐induced ONFH. These EVs localized to the femoral head and supported vascular and bone regeneration. In contrast, pathological EVs from diseased or aged tissues may promote ONFH. For example, EVs from patients with systemic mastocytosis were enriched in miR‐23a and miR‐30a, which inhibited RUNX2 and SMAD1/5, suppressed osteogenesis, and induced trabecular bone loss in vivo [59]. Similarly, EVs from aged bone matrix were enriched in miR‐483‐5p and miR‐2861, promoting adipogenic transdifferentiation of BMSCs and osteogenic conversion of vascular smooth muscle cells, contributing to the “osteoporosis‐vascular calcification paradox” [60]. Altogether, ONFH emerges as a complex disease driven by a dysregulated EV network. GC exposure depletes beneficial microbial EVs and enriches pathological EVs, jointly impairing osteogenesis, angiogenesis, and immune homeostasis. Our study underscores the therapeutic potential of engineered miR‐146a‐5p‐EVs, which not only restore protective miRNAs but also counteract pathological EV signaling. The EV‐mediated interaction between gut microbiota and bone, analogous to MSC‐derived EV communication, offers a new paradigm for inter‐organ metabolic regulation.

Finally, our results highlight the therapeutic potential of targeting miR‐146a‐5p and EV signaling in GC‐ONFH. Currently, effective disease‐modifying treatments for GC‐induced ONFH remain limited, and most patients ultimately require total hip arthroplasty [1]. In contrast, exosome‐ or miRNA‐based therapies offer a novel approach for intervention at disease‐relevant stages and comprehensive treatment. For example, MSC‐derived EVs have been reported to alleviate GC‐induced osteonecrosis by enhancing endothelial function, restoring osteogenic differentiation, and suppressing inflammatory cytokine production. These findings support EVs as effective mediators of intercellular communication in femoral head repair, although efficient local retention and targeted delivery remain key challenges [61, 62]. In parallel, dysregulated miRNA networks have been implicated in ONFH pathogenesis, regulating inflammation, oxidative stress, osteogenic commitment, and cell survival. Multiple miRNAs have been shown to influence GC‐induced bone injury by targeting inflammatory signaling cascades or osteogenic regulators [27, 63, 64]. However, the therapeutic application of free miRNAs is limited by rapid degradation, insufficient tissue targeting, and transient bioactivity in vivo.

Our study integrates these two lines of research by employing engineered EVs as a delivery vehicle for miR‐146a‐5p, thereby overcoming key limitations of miRNA therapy while leveraging the intrinsic reparative properties of EVs. Unlike prior studies focusing on either EVs or miRNAs alone, our approach combines delivery optimization with coordinated pathway regulation. Through localized intraosseous administration, engineered EVs achieve enhanced retention within the femoral head region, maximizing bioavailability while minimizing systemic exposure. Mechanistically, exosomal miR‐146a‐5p exerts multi‐pathway effects by attenuating inflammatory signaling and restoring mitochondrial quality control, including autophagy‐associated processes, thereby rescuing BMSC function under GC stress. This dual advantage, optimized delivery and synergistic regulation of inflammatory and mitochondrial stress pathways, distinguishes our strategy from existing EV‐ or miRNA‐based interventions in ONFH. Future studies should address optimal delivery methods, dosing, and long‐term safety. Despite promising prospects, several critical challenges remain. Long‐term efficacy and safety require rigorous preclinical and clinical validation. Further research is needed on EVs uptake mechanisms, miRNA processing, and their metabolic and immune effects on recipient cells. Additionally, improving in vivo targeting to femoral head lesions, developing scalable manufacturing processes for engineered EVs, and evaluating differential therapeutic responses across disease stages or patient subgroups are key for clinical translation.

In summary, our study identified miR‐146a‐5p as a central molecular integrator of inflammatory signaling, mitochondrial dysfunction, and stem cell senescence in GC‐induced ONFH. By leveraging engineered exosomes for targeted delivery, we demonstrated that restoration of miR‐146a‐5p efficiently suppresses the TRAF6‐NF‐κB pathway, alleviates oxidative stress, preserves mitochondrial homeostasis, and reactivates both autophagic and mitophagic flux in compromised BMSCs. As a result, exosomal miR‐146a‐5p therapy not only rejuvenates BMSC function at both cellular and transcriptomic levels but also promotes global remodeling of the bone marrow niche, coordinating osteogenic and angiogenic regeneration. Collectively, these findings establish miR‐146a‐5p‐EVs as a promising, biologically compatible, and cell‐free therapeutic strategy to restore skeletal regeneration in the context of pathological glucocorticoid exposure.

### 3.1 Limitations of the Study

Future studies should address optimal delivery methods, dosing, and long‐term safety. Despite promising prospects, several critical challenges remain. Long‐term efficacy and safety require rigorous preclinical and clinical validation. Further research is needed on EVs uptake mechanisms, miRNA processing, and their metabolic and immune effects on recipient cells. Additionally, improving in vivo targeting to femoral head lesions, developing scalable manufacturing processes for engineered EVs, and evaluating differential therapeutic responses across disease stages or patient subgroups are key for clinical translation.

### 3.2 Significance Statement

GC‐induced osteonecrosis remains a major clinical challenge, driven by intertwined mechanisms of inflammation, mitochondrial dysfunction, and stem cell exhaustion. Our work identified miR‐146a‐5p as a key regulator that can simultaneously reverse these pathogenic pathways. Utilizing engineered exosomes for precise miR‐146a‐5p delivery, we provided a targeted approach to suppress inflammation, restore mitochondrial health, and rejuvenate stem cell function. This multi‐pronged strategy not only enhanced bone regeneration and angiogenesis in clinically relevant models but also established a versatile framework for cell‐free therapy, with broad applicability to other metabolic, inflammatory, and degenerative diseases. Our findings highlighted the translational promise of miRNA‐engineered exosome therapeutics for musculoskeletal and systemic tissue regeneration.

## 4 Materials and Methods

### 4.1 Human Sample Collection

Bone marrow samples were obtained intraoperatively from patients undergoing total hip arthroplasty at Peking Union Medical College Hospital (Beijing, China). Patients in the osteonecrosis of the femoral head (ONFH) group had systemic lupus erythematosus (SLE) and a documented history of glucocorticoid use. Controls were patients with developmental dysplasia of the hip (DDH) who had no history of glucocorticoid exposure. All donors provided informed consent in accordance with the guidelines approved by the Ethics Committee of Peking Union Medical College Hospital (Approval No. JS‐2625).

### 4.2 Rat Model Construction and Treatment

Male Sprague‐Dawley rats (8 weeks old, ∼250g) were obtained from Beijing Vital River Laboratory Animal Technology Co., Ltd and housed under SPF conditions. ONFH was induced by intraperitoneal injection of lipopolysaccharide (LPS, 20µg/kg) followed 24h later by MPS (40mg/kg/day) for 3 consecutive days, as previously described. Control animals received saline injections. All animal procedures were approved by the Institutional Animal Care and Use Committee of Peking Union Medical College Hospital (Approval No. JS‐2625).

One week after ONFH induction, animals were anesthetized with isoflurane and placed in the lateral decubitus position. MiR‐146a‐5p‐enriched exosomes (miR‐146a‐5p‐EVs, 100µg in 50µL PBS) or control EVs were injected into the femoral head using a Hamilton microsyringe under stereotactic guidance. Injections were performed once weekly for 3 weeks. Rats were sacrificed for analysis 8 weeks after the first injection.

### 4.3 Whole Transcriptome Sequencing

Total RNA from cultured bone marrow mesenchymal stem cells (BMSCs) was extracted using TRIzol reagent (Thermo Fisher Scientific) and assessed for quality and integrity using an Agilent 2100 Bioanalyzer. Library preparation and sequencing were performed by Novogene Co., Ltd. (Beijing, China). Ribosomal RNA was removed for mRNA/lncRNA sequencing using the NEBNext rRNA Depletion Kit, and libraries were constructed using the NEBNext Ultra RNA Library Prep Kit (NEB). For miRNA profiling, libraries were generated using the NEBNext Multiplex Small RNA Library Prep Kit. Sequencing was performed on an Illumina NovaSeq 6000 platform. Raw reads were quality‐filtered and mapped to the human genome (GRCh38) using HISAT2 (v2.1.0) for mRNA/lncRNA and Bowtie (v1.2.3) for miRNA. Expression levels were quantified with feature Counts and normalized to TPM.

### 4.4 Competing Endogenous RNA (ceRNA) Network Analysis

ceRNA networks centered on miR‐146a‐5p were constructed by integrating expression profiles and target predictions from miRANda and TargetScan. Correlation coefficients (Pearson r <0.7, *p* < 0.05) were used to identify meaningful interactions. The network was visualized using Cytoscape software (v3.8.2).

### 4.5 Quantitative Real‐Time Polymerase Chain Reaction (RT‐qPCR)

RT‐qPCR was performed to validate miRNA expression. Total RNA was transcribed in reverse using the PrimeScript RT reagent Kit (Takara), and amplification was conducted using TB Green Premix Ex Taq (Takara) on the ABI Prism 7500 system (Applied Biosystems, CA, USA). miR‐146a‐5p expression was normalized to U6 snRNA. Relative expression was calculated using the 2^−ΔΔCt^ method. Each reaction was run in triplicate.

### 4.6 Serum miRNA Sequencing

Peripheral blood samples were collected from three groups, including SLE patients with ONFH, SLE patients without ONFH, and DDH controls. Serum was isolated, and total RNA was extracted using the miRNeasy Serum/Plasma Kit (Qiagen). Small RNA library preparation and sequencing were performed by LC‐Bio Technology Co., Ltd. (Hangzhou, China) using the Illumina HiSeq platform with single‐end 50 bp reads. Data processing and differential expression analyses followed the same pipeline as described above.

### 4.7 Engineering of miR‐146a‐5p‐Enriched Exosomes (miR‐146a‐5p‐EVs)

Exosomes were first isolated from the conditioned medium of healthy BMSCs as previously described. For miRNA loading, exosomes (20µg total protein) were mixed with synthetic miR‐146a‐5p mimic (RiboBio) in electroporation buffer (Thermo Fisher Scientific) and subjected to electroporation using a Gene Pulser Xcell Electroporation System (Bio‐Rad) at 400V, 125µF. Samples were incubated on ice post‐electroporation and washed with PBS to remove free miRNA.

Transmission electron microscopy (TEM) was performed to assess exosome morphology after electroporation. Nanoparticle tracking analysis (NTA; Malvern) was used to determine particle size distribution. Expression of exosomal protein markers (ALIX, HSP70, and TSG101) was confirmed by Western blot.

### 4.8 Exosome Uptake Assay

To visualize uptake, exosomes were labeled with Dil (Thermo Fisher Scientific) following the manufacturer's protocol and incubated with ONFH‐BMSCs for 24h. Cells were fixed with 4% paraformaldehyde, stained with DAPI, and imaged using a confocal laser scanning microscope to assess the intracellular localization of labeled exosomes.

### 4.9 Functional Rescue Assays

ONFH‐BMSCs were treated with either miR‐146a‐5p‐EVs or control exosomes (Ctrl‐EVs) at a concentration of 20µg/mL for 48h. After treatment, cells were induced for osteogenic or adipogenic differentiation. Oil red O staining was performed to evaluate adipogenesis. Senescence was assessed using SA‐β‐galactosidase staining. ALP activity staining (day 7) and Alizarin Red S staining (day 14) were performed to evaluate osteogenic differentiation.

### 4.10 Wound Healing Assay (Scratch Migration)

Human umbilical vein endothelial cells (HUVECs) were seeded into 6‐well plates until 90% confluency, followed by a linear scratch using a 200µL pipette tip. After washing to remove detached cells, fresh medium containing Ctrl‐EVs or miR‐146a‐5p‐EVs was added. Images were taken at 0 and 24h using a phase‐contrast microscope.

### 4.11 Transwell Migration Assay

HUVECs (1 × 10^5^ cells/well) were seeded into the upper chamber of a transwell insert (8µm pore size; Corning) in serum‐free ECM, with exosome‐containing medium in the lower chamber. After 12h incubation, migrated cells on the lower membrane surface were fixed, stained with crystal violet, and counted under a microscope.

### 4.12 Tube Formation Assay

Matrigel (Corning) was thawed at 4°C and added to a 96‐well plate (50 µL/well), polymerized at 37°C for 30min. Treated HUVECs (2 × 10^4^ cells/well) were seeded onto the Matrigel and incubated for 4–6h. Capillary‐like structures were imaged under a light microscope, and quantitative analysis of nodes, junctions, and tube lengths was performed using ImageJ with the Angiogenesis Analyzer plugin.

### 4.13 Immunofluorescence Staining

Treated HUVECs were fixed with 4% paraformaldehyde, permeabilized with 0.1% Triton X‐100, blocked in 5% BSA, and incubated with primary antibodies against von Willebrand factor (vWF, 1:100, Abcam) and CD31 (1:100, Abcam) overnight at 4°C. After washing, cells were incubated with Alexa Fluor‐conjugated secondary antibodies, counterstained with DAPI, and imaged using a confocal microscope.

### 4.14 Gait Analysis

Motor function was assessed using the DigiGait treadmill (Mouse Specifics, Inc., USA). Rats were trained prior to recording. Parameters were measured, including stand time, swing time, stride frequency, and stride length. Data were averaged from at least three consistent runs per animal.

### 4.15 Micro‐CT Scanning and Quantification

After euthanasia, femoral heads were fixed in 4% paraformaldehyde and scanned using a high‐resolution micro‐CT system (Siemens) with scanning parameters of 50kV, 500µA, and 9µm resolution. 3D reconstruction and morphometric analyses were conducted using CTAn software. The quantitative indices obtained included trabecular number (Tb.N), trabecular thickness (Tb.Th), trabecular separation (Tb.Sp), and bone volume fraction (BV/TV).

### 4.16 Histology and Histomorphometry

Decalcified femoral heads were embedded in paraffin and sectioned at 5µm. Sections were stained with hematoxylin and eosin (H&E) for structural assessment and Masson's trichrome for collagen deposition. Images were acquired using a digital slide scanner (3D histech, 3DHISTECH).

### 4.17 Dynamic Bone Formation Labeling

Calcein double labeling was performed to assess bone formation rates in vivo. Rats were intraperitoneally injected with calcein (20mg/kg; Sigma‐Aldrich) on days 10 and 3 before euthanasia. Undecalcified femoral heads were embedded in polymethyl methacrylate (PMMA) and sectioned at 10µm using a microtome. Fluorescent labeling was imaged under a fluorescence microscope, and the mineral apposition rate (MAR) was calculated as the distance between two calcein labels divided by the interval (µm/day) using ImageJ.

### 4.18 Microfil Vascular Perfusion

To visualize intraosseous vasculature, rats were anesthetized and perfused via the abdominal aorta with heparinized saline, followed by 4% paraformaldehyde and then Microfil compound (Flow Tech Inc) at physiological pressure. Femurs were harvested, fixed overnight, and scanned using micro‐CT at 9µm resolution. 3D vascular reconstruction and vessel morphometry were performed using CTAn and CTVol software.

### 4.19 Immunofluorescence for Bone and Vascular Markers

Decalcified paraffin‐embedded femoral head sections (5µm) were used for double immunofluorescence staining. After antigen retrieval and blocking with 5% BSA, sections were incubated overnight at 4°C with primary antibodies against COL1A1 (1:100, Abcam) and vWF (1:100, Abcam). Alexa Fluor 488‐ or 594‐conjugated secondary antibodies (Invitrogen) were applied for detection. Nuclei were counterstained with DAPI, and imaging was performed on a confocal microscope. Fluorescence analysis was performed using ImageJ to evaluate the relative fluorescence intensity of osteogenic and angiogenic signals.

### 4.20 Single‐Cell RNA Sequencing (scRNA‐seq)

Sample preparation: Femoral heads were collected from ONFH rats treated with PBS or miR‐146a‐5p‐EVs and immediately subjected to enzymatic dissociation. The bones were minced and digested in collagenase type I (2mg/mL) and DNase I (100U/mL) at 37°C for 45 min with agitation. The cells were filtered through a 70µm strainer, resuspended in PBS with 0.04% BSA, and cell viability (>85%) was confirmed using trypan blue exclusion. The samples were then loaded into the 10x Genomics Chromium Controller for single‐cell barcoding using the Chromium Single Cell 3’ v3.1 Reagent Kit.

Library preparation and sequencing: Library construction followed the 10x Genomics user guide. cDNA was amplified and sequenced on an Illumina NovaSeq 6000 platform to obtain a minimum depth of 50 000 reads per cell. The raw sequencing data were processed using Cell Ranger (v6.0.1) and aligned to the Rattus norvegicus reference genome (Rnor_6.0).

Data processing and clustering: The gene‐cell count matrix was filtered to remove cells with fewer than 200 or more than 5000 detected genes or with mitochondrial gene content exceeding 15%. Data normalization, variable gene identification, PCA, clustering (resolution = 0.5), and UMAP visualization were performed using Seurat (v4.1.0) in R. Cell clusters were annotated based on canonical markers for various cell types.

Differential expression and functional analysis: Marker gene expression across conditions was visualized using FeaturePlot and DotPlot functions in Seurat. Differential gene expression was analyzed using the Wilcoxon rank‐sum test (adjusted p < 0.05), retaining genes with ≥0.25 log fold‐change and expressed in ≥10% of cells. BMSC subclusters were further analyzed using FindSubCluster, revealing four major functional states. Proportional changes in subclusters were compared between groups using the propeller function in the speckle R package.

### 4.21 RNA Sequencing and Analysis

Library construction and sequencing were performed by OE Biotech Co., Ltd. (Shanghai, China). Briefly, mRNA was enriched through poly(A) selection from total RNA. The poly(A)+ RNA was then fragmented to 150 bp and converted to cDNA. The cDNA fragments were end‐repaired, adenylated, adapter‐ligated, and amplified by PCR. The final libraries were sequenced on the Illumina NovaSeq 6000 platform with 2 × 150 bp paired‐end sequencing, ensuring a minimum of 20 million reads per sample.

Raw reads were trimmed using Trimmomatic and aligned to the rat reference genome (Rnor_6.0) with HISAT2 (v2.1.0). Gene expression was quantified using featureCounts, and differential expression analysis was conducted with DESeq2 (v1.30.1). Gene Ontology (GO) and KEGG pathway enrichment analyses were performed using ClusterProfiler (v3.18.1), focusing on GO terms related to osteogenesis, ECM remodeling, oxidative phosphorylation, and mitochondrial metabolism, as well as KEGG pathways involved in ribosome biogenesis, metabolic activation, and inflammation suppression. All analyses were performed using R (4.1.2) and R Studio (1.4.1106).

### 4.22 Target Prediction and Dual‐Luciferase Reporter Assay

Potential miR‐146a‐5p targets were identified using TargetScan, miRanda, and miRDB, all of which predicted TRAF6 as a conserved downstream target. The 3’ untranslated region (UTR) of TRAF6 containing the predicted miR‐146a‐5p binding site was cloned into the psiCHECK‐2 vector (Promega). A mutant construct with base substitutions in the seed‐binding region was also generated. HEK293T cells were co‐transfected with either wild‐type or mutant reporters and miR‐146a‐5p mimic or negative control using Lipofectamine 2000. Luciferase activity was measured 48h later using the dual‐luciferase reporter assay system (Promega), and Renilla/firefly ratios were calculated.

### 4.23 Western blot

Western blotting was performed to assess protein expression in BMSCs. Total protein was extracted using RIPA buffer supplemented with protease and phosphatase inhibitors. Protein samples (30µg) were separated by SDS‐PAGE and transferred to PVDF membranes. The membranes were incubated with primary antibodies, including TRAF6 (1:2000, Abcam), NF‐κB p65 (phospho S536, 1:2000, Abcam), total NF‐κB p65 (1:2000, Abcam), β‐actin (1:2000, Abcam) for the general analysis and Beclin1 (1:2000, Abcam), ATG5 (1:2000, Abcam), ATG4B (1:2000, Abcam), LC3B (1:2000, Abcam) and p62 (1:2000, Abcam) for autophagy‐related proteins. Detection was performed using HRP‐conjugated secondary antibodies and ECL substrate (Bio‐Rad). The ratio of LC3B‐II to LC3B‐I was calculated to assess autophagosome formation, and p62 accumulation was used as an indicator of impaired autophagic flux.

### 4.24 Mitochondrial Function Detection

Mitochondrial membrane potential was assessed using JC‐1 dye (Yisheng). BMSCs were stained with JC‐1 (5 µg/mL) at 37°C for 20 min. Red fluorescence, representing JC‐1 aggregates, indicated healthy mitochondria, while green fluorescence, corresponding to JC‐1 monomers, indicated depolarized mitochondria.

Reactive oxygen species (ROS) levels were measured using DCFH‐DA (10 µM, Beyotime). Cells were incubated at 37°C for 30 min, washed, and immediately imaged by fluorescence microscopy to detect intracellular ROS.

Mitochondrial respiration was assessed by measuring the oxygen consumption rate (OCR) of BMSCs seeded in Seahorse XF96 plates and treated with miR‐146a‐5p‐EVs or control for 48 h. The Seahorse XF Cell Mito Stress Test Kit (Agilent) was used to measure basal respiration, maximal respiration, and spare respiratory capacity following sequential injection of oligomycin, FCCP, and rotenone/antimycin A.

Mitochondrial morphology was examined by TEM (JEOL) after fixing BMSCs in 2.5% glutaraldehyde, post‐fixing in osmium tetroxide, dehydration, resin embedding, and ultrathin sectioning. Additionally, mitochondrial length and network integrity were further assessed by confocal microscopy after MitoTracker (Yisheng) staining. Structured illumination microscopy (SIM) was performed after staining BMSCs with PK Mito Deep Red (100nM; Warbio).

### 4.25 Immunofluorescence for Autophagy and Mitophagy

Cells were fixed and immunostained with the following combinations: TOM20 (1:200, Abcam) as a mitochondrial outer membrane marker, LC3B (1:200, Abcam) as an autophagosome marker, and LAMP1 (1:200, Abcam) as a lysosomal marker. After incubation with Alexa Fluor secondary antibodies, nuclei were counterstained with DAPI.

To evaluate autophagic flux, BMSCs were transfected with a mCherry‐GFP‐LC3B adenovirus (Beyotime). After 24 h, cells were treated with miR‐146a‐5p‐EVs or PBS for an additional 24 h, followed by fixation and imaging with confocal microscopy.

### 4.26 Statistical Analysis

Statistical analyses were performed using GraphPad Prism 9 and R (v4.0.3). Data are presented as mean ± standard error of the mean (SEM). Unless otherwise stated, in vitro experiments were performed using three independent biological replicates per group (n = 3), which is consistent with standard practice in mechanistic cell‐based studies. For Seahorse metabolic flux analyses, six independent biological replicates per group (n = 6) were used to account for higher measurement variability. Animal experiments were conducted with four animals per group (n = 4), as determined based on prior studies and preliminary data. Where applicable, each biological replicate was measured in triplicate (technical replicates). Comparisons between two groups were performed using two‐tailed unpaired Student's t‐tests, while comparisons among more than two groups were conducted using one‐way ANOVA followed by Tukey's post‐hoc test.

## Author Contributions

Z.L and X.C. jointly conceived, designed and executed the experimental work of this study, including sequencing data processing, miRNA screening, exosome handling, treatment validation and wrote the manuscript draft. Y.X. assisted in collecting patient bone marrow and serum samples. X.Y. assisted with cell and animal experiments. R.W. contributed to data analysis. H.W. assisted in data organization and statistical analysis, while Y.B. and J.X. assisted with manuscript revisions. J.L. and L.L. provided platform technical support and experimental equipment. Y.W., B.F., J.S. and X.W. were responsible for overall project supervision, funding support, and manuscript revisions. All authors contributed to the manuscript revisions and feedback.

## Funding

This work is funded by the National Natural Science Foundation of China (Grant ID: 82572715 and 82302708), the National Key Research and Development Program of China (2018YFE0104200), the Beijing Natural Science Foundation (Grant ID: 7232129), and CAMS Innovation Fund for Medical Sciences (CIFMS) (Grant ID: 2025‐I2M‐XHXX‐014), Beijing Outstanding Young Scientist Program (JWZQ20240101010), and the National Natural Science Foundation of China (No. U22A20348).

## Conflicts of Interest

The authors declare no conflicts of interest.

## Supporting information




**Supporting File**: advs75897‐sup‐0001‐SuppMat.docx.

## Data Availability

The data that support the findings of this study are available on request from the corresponding author. The data are not publicly available due to privacy or ethical restrictions.
