# Supplementary material for: Engineered Exosomal miR‐146a‐5p Reprograms BMSC Fate and Restores Mitochondrial Homeostasis in Glucocorticoid‐Induced Osteonecrosis of Femoral Head
Source: Adv Sci (Weinh). 2026 Jun 15:e75897. Online ahead of print. doi: 10.1002/advs.75897 (PMC13336745; doi:10.1002/advs.75897)
Supplement: Supplementary file 1 — Supporting File: advs75897‐sup‐0001‐SuppMat.docx. [file ADVS-9999-e75897-s001.docx]

**Supplemental information**

**Engineered Exosomal miR-146a-5p Reprograms BMSC Fate and Restores Mitochondrial Homeostasis in Glucocorticoid-Induced Osteonecrosis of Femoral Head**

**Zehui Lv, Xuejie Cai, Yiming Xu, Xingdong Yang, Ruoying Wang, Han Wang, Yixin Bian, Jiawei Xu,Jiao Lu, Lulu Liu, Yingjie Wang, Jibin Song, Bin Feng, and Xisheng Weng**


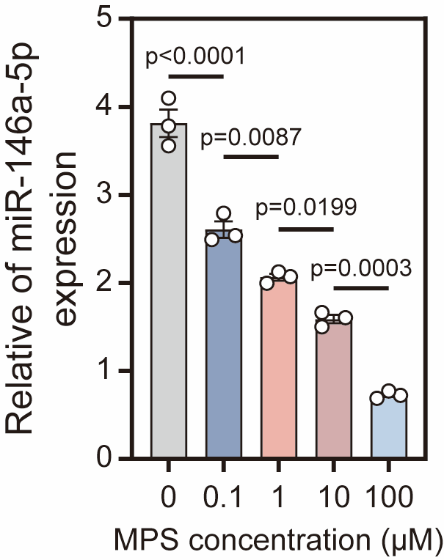


**Figure S1.** Dose-dependent suppression of miR-146a-5p by methylprednisolone (MPS) in BMSCs (n=3). Data are presented as mean ± SEM. One-way ANOVA with Tukey’s post hoc test was used for multiple comparisons. Exact P values are reported in the figure.


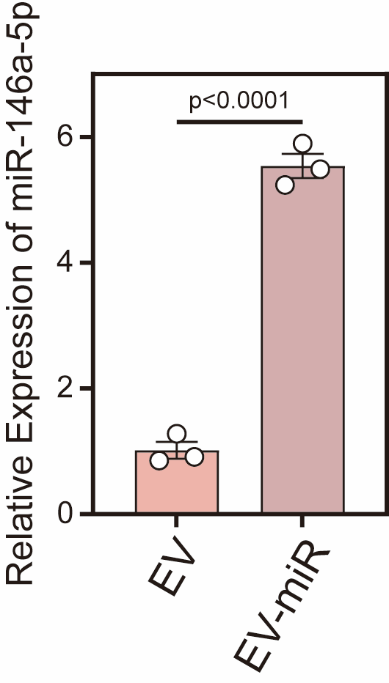


**Figure S2.** Expression level of miR-146a-5p in extracellular vesicles (n=3). Data are presented as mean ± SEM. Two-tailed Student’s t-test was used for comparison. Exact P values are reported in the figure.


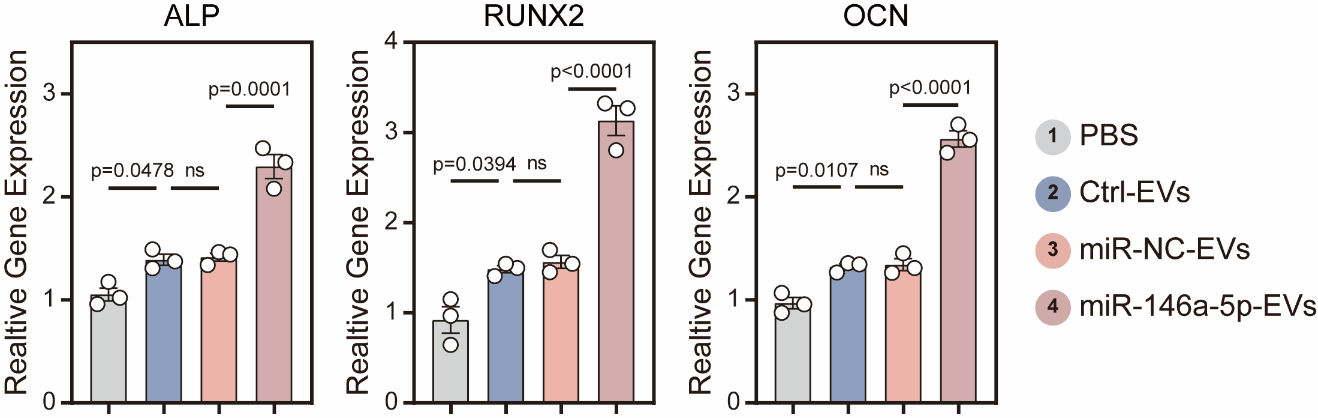


**Figure S3.** RT-qPCR analysis of osteogenic gene (ALP, RUNX2 and OCN) expression (n=3). Data are presented as mean ± SEM. One-way ANOVA with Tukey’s post hoc test was used for multiple comparisons. Exact P values are reported in the figure; ns, not significant.


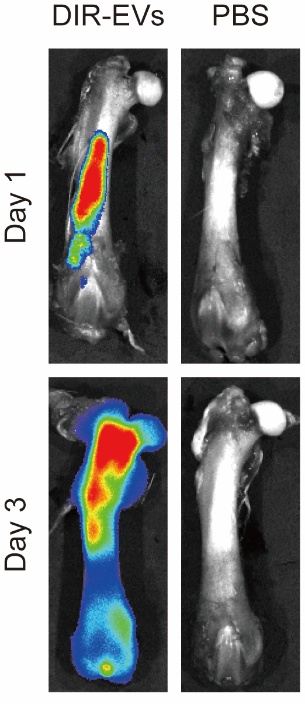


**Figure S4.** Ex vivo fluorescence imaging of isolated femora after intraosseous injection of DIR-labeled exosomes or PBS.

**
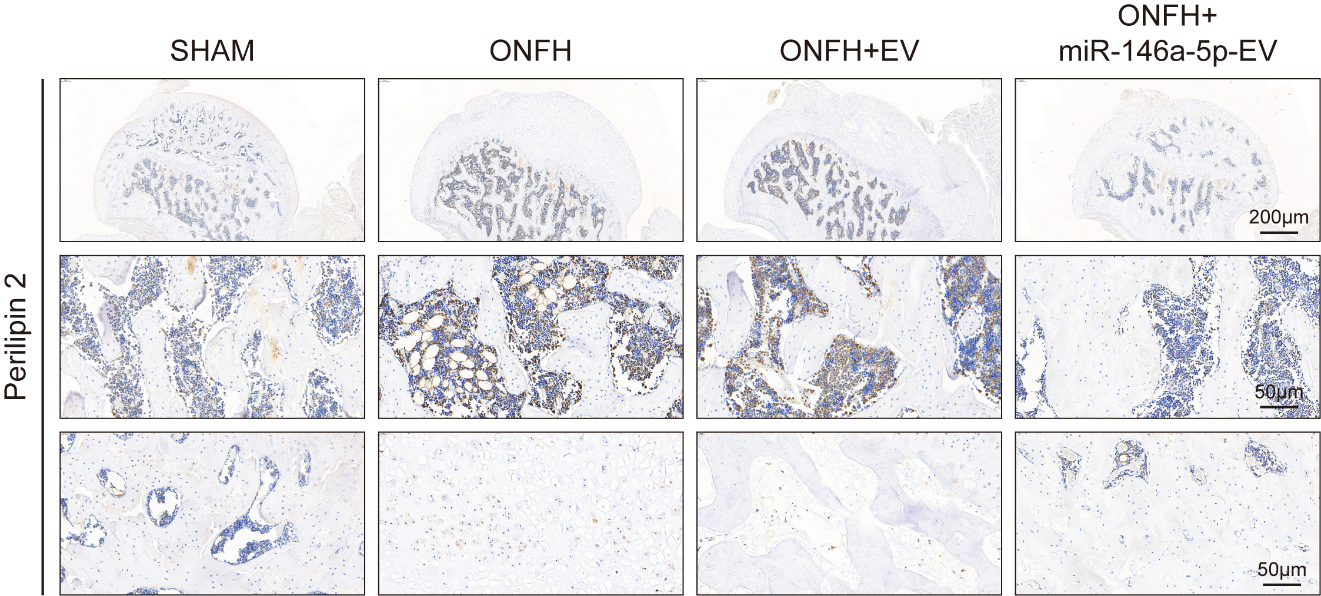
**

**Figure S5.** Immunohistochemical staining of Perilipin 2 was performed in all groups


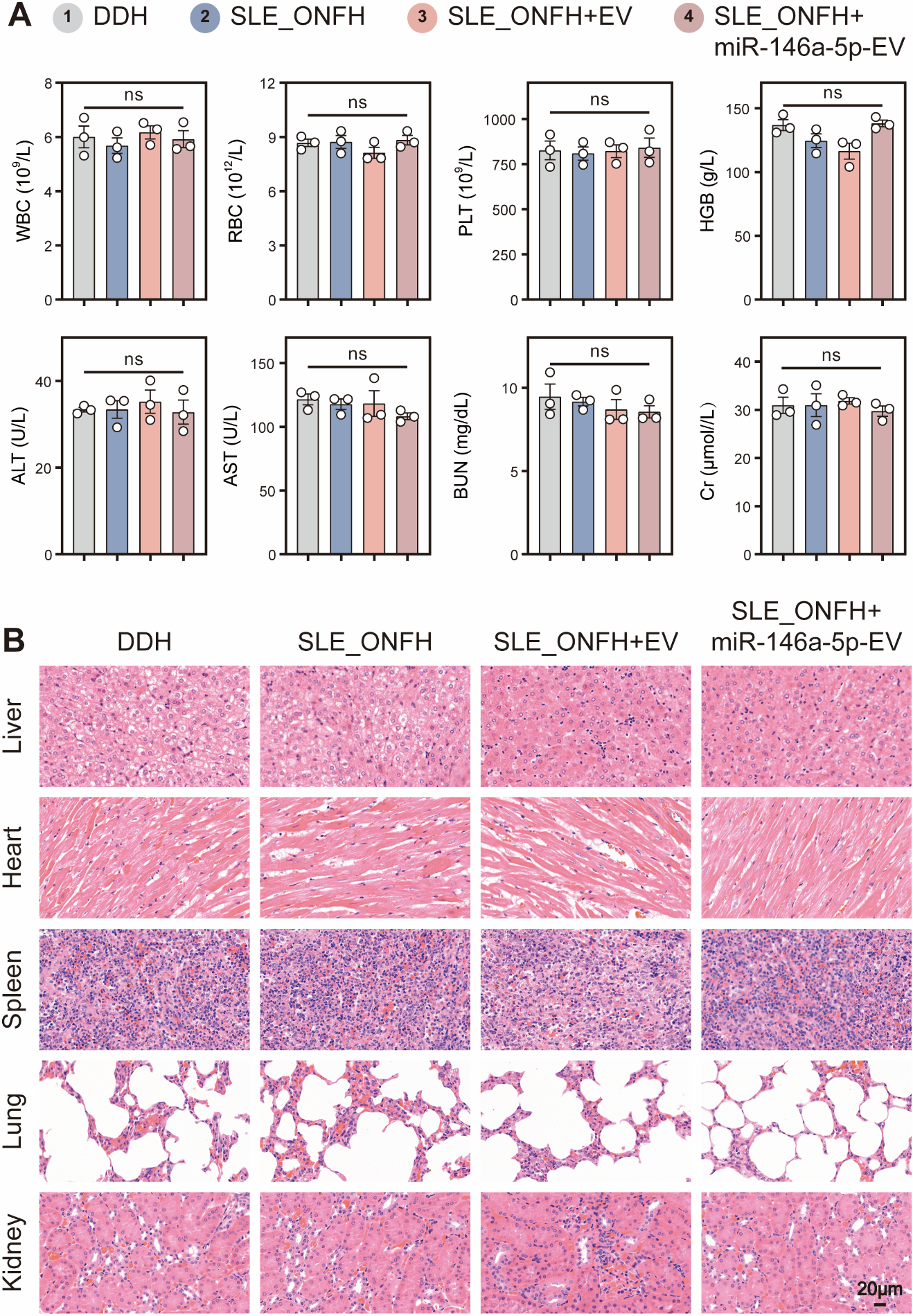


**Figure S6.** In vivo biosafety evaluation of engineered exosome treatment.

(A) Blood routine and serum biochemical parameters showing no significant systemic toxicity after treatment (n=3). Data are presented as mean ± SEM. One-way ANOVA with Tukey’s post hoc test was used for multiple comparisons. ns, not significant.

(B) H&E staining of major organs (liver, heart, spleen, lung, kidney).


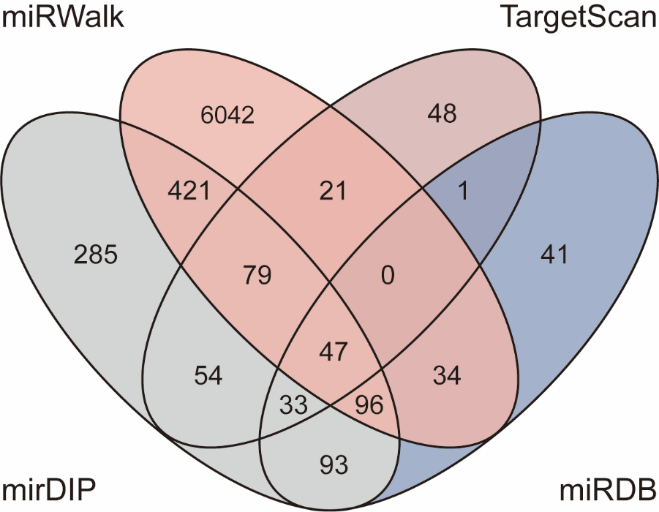


**Figure S7.** Prediction intersection of four databases.


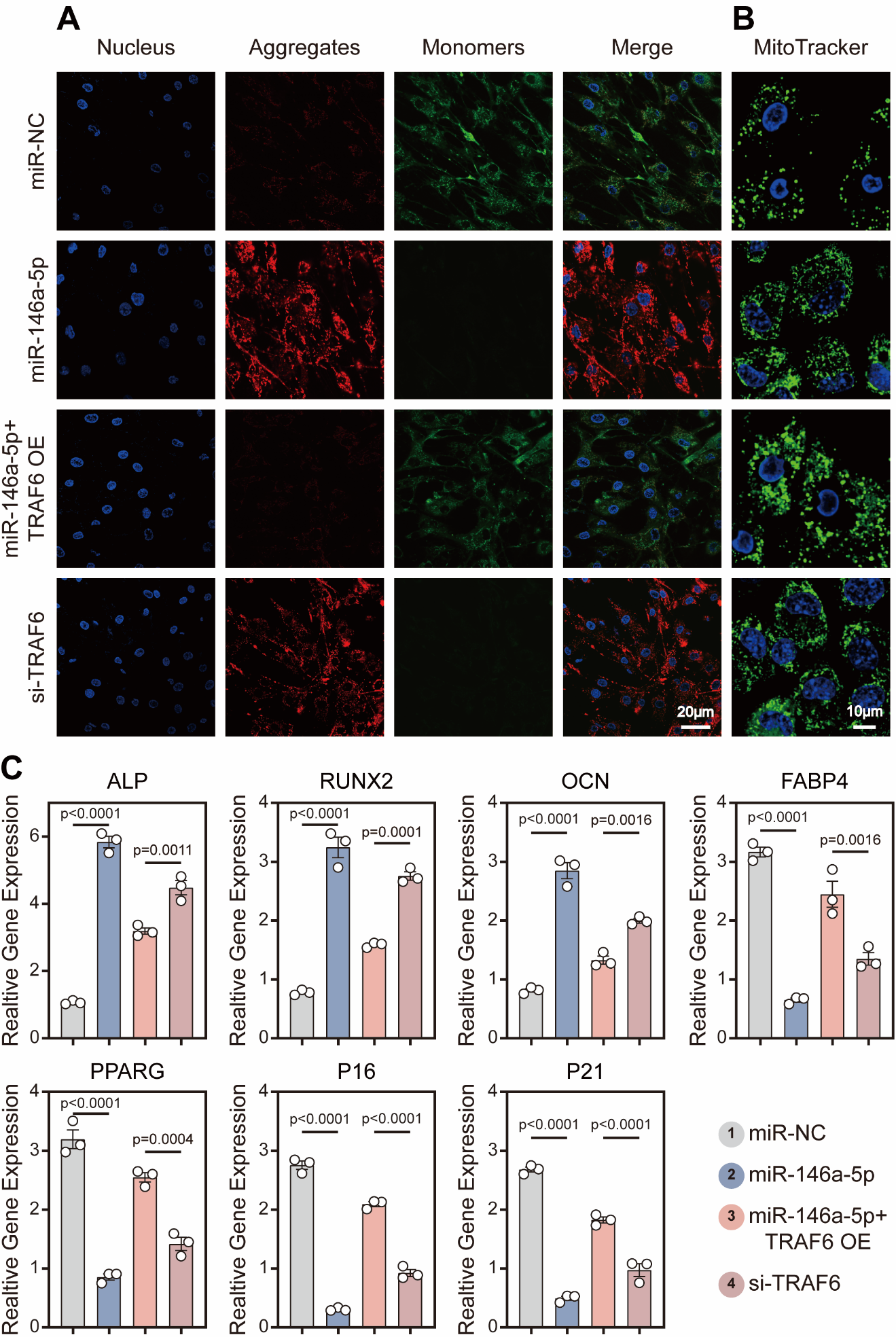


**Figure S8.** miR-146a-5p regulates mitochondrial homeostasis and BMSC fate through TRAF6 in ONFH-derived BMSCs.

(A) Mitochondrial membrane potential was evaluated by JC-1 staining.

(B) Mitochondrial status was assessed by MitoTracker staining.

(C) Relative expression of osteogenic, adipogenic, and senescence-related genes was analyzed by qPCR (n=3). Data are presented as mean ± SEM. One-way ANOVA with Tukey’s post hoc test was used for multiple comparisons. Exact P values are reported in the figure.


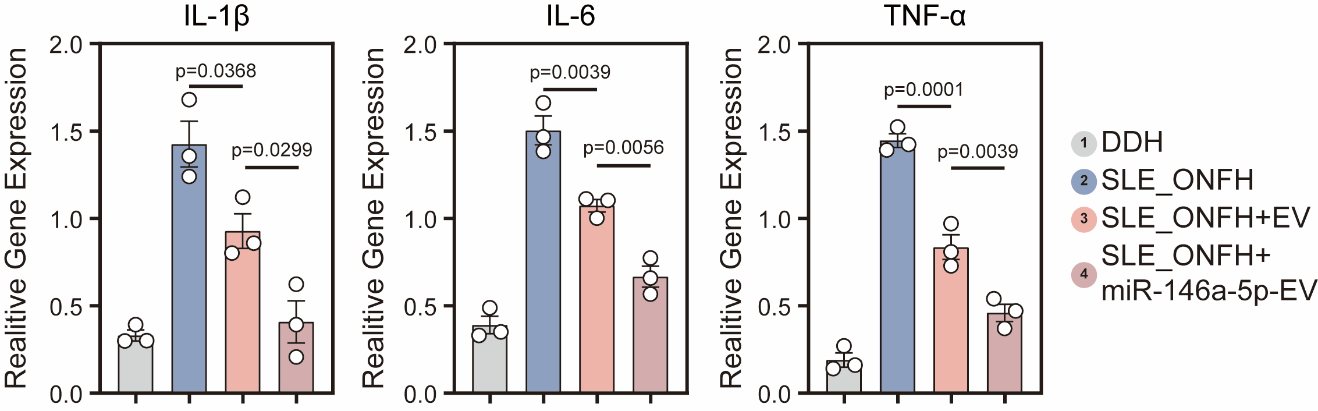


**Figure S9.** RT-qPCR analysis of NF-κB downstream target genes (IL-1β, TNF-α, and IL-6) (n=3). Data are presented as mean ± SEM. One-way ANOVA with Tukey’s post hoc test was used for multiple comparisons. Exact P values are reported in the figure.


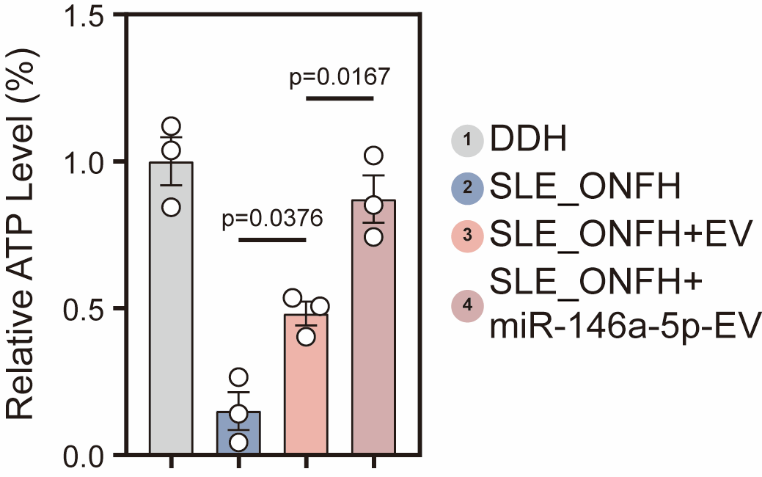


**Figure S10.** ATP level of different treatments (n=3). Data are presented as mean ± SEM. One-way ANOVA with Tukey’s post hoc test was used for multiple comparisons. Exact P values are reported in the figure.


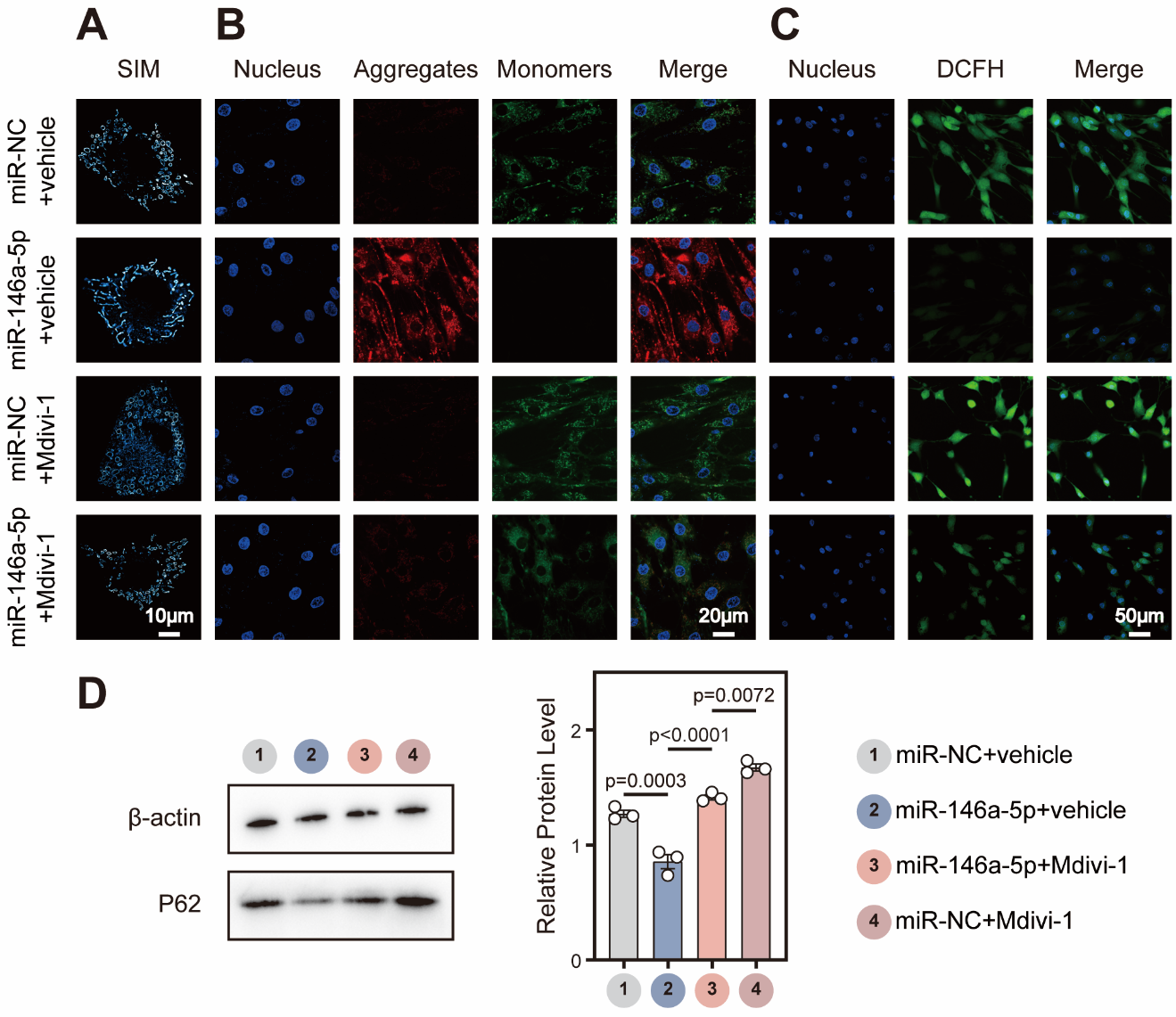


**Figure S11.** Mitophagy inhibition attenuates the mitochondrial protective effects of miR-146a-5p in ONFH-BMSCs.

(A) Super-resolution structured illumination microscopy (SIM) images showing mitochondrial morphology in ONFH-BMSCs under the indicated treatments.

(B) Mitochondrial membrane potential was evaluated by JC-1 staining.

(C) Intracellular ROS levels in ONFH-BMSCs.

(D) Western blot and quantitative analysis of P62 expression (n=3). Data are presented as mean ± SEM. One-way ANOVA with Tukey’s post hoc test was used for multiple comparisons. Exact P values are reported in the figure.

| miR_name | p-value (ANOVA) |
| --- | --- |
| hsa-miR-21-5p | 0.0003616993 |
| hsa-miR-199a-3p | 0.0006890184 |
| hsa-miR-625-3p | 0.0015304673 |
| hsa-mir-10401-p5 | 0.0016585269 |
| hsa-miR-6741-5p_R+1 | 0.0026234085 |
| PC-5p-181243_78 | 0.0116762913 |
| hsa-miR-146a-5p | 0.0174744301 |
| hsa-miR-151a-3p | 0.0175351724 |
| PC-3p-166086_88 | 0.0201787247 |
| hsa-miR-6875-5p | 0.02057007 |
| hsa-miR-556-3p_R-1 | 0.0252398032 |
| hsa-miR-483-3p_L-1R+2 | 0.0377218267 |
| hsa-miR-1270 | 0.0421175641 |
| hsa-miR-224-5p_L-1 | 0.044509168 |

**Table S1.** List of 14 intersecting miRNAs.


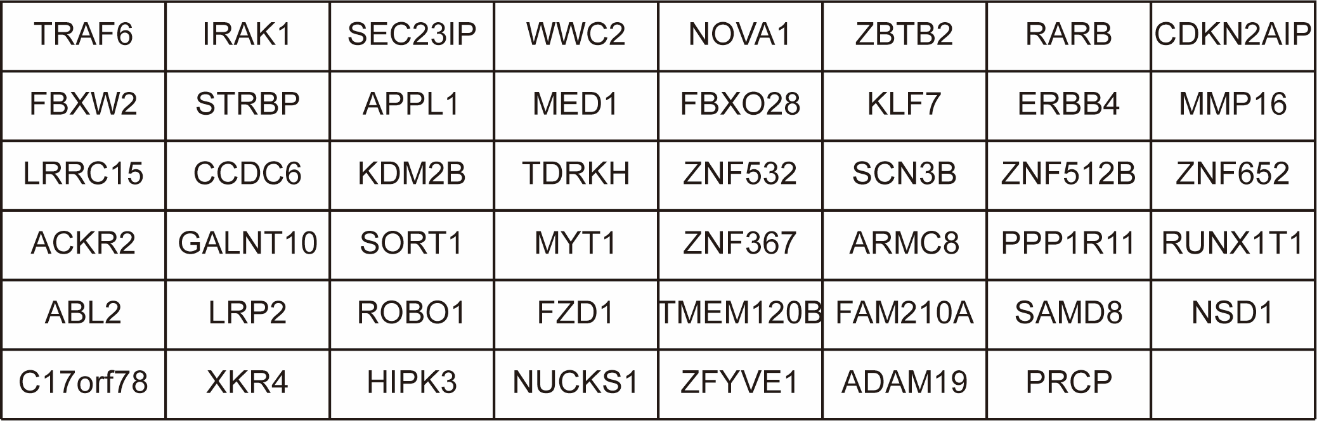


**Table S2.** Predicted mRNA targets of miR-146a-5p identified across multiple databases.


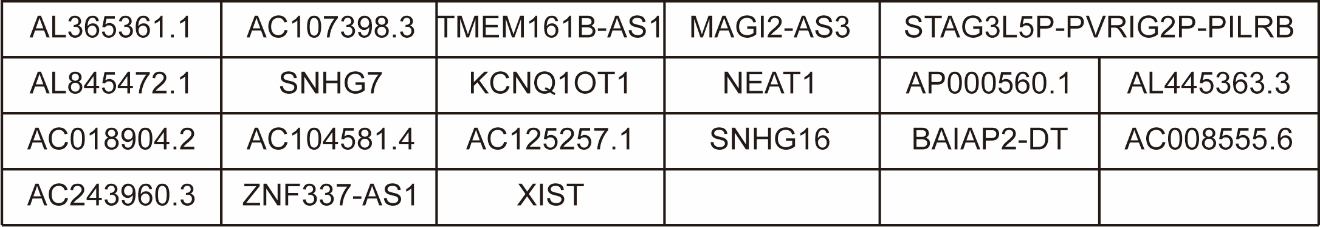


**Table S3.** Predicted lncRNA interactors of miR-146a-5p identified from ENCORI.

| Gene | Primer | Sequence |
| --- | --- | --- |
| hsa-GAPDH  hsa-ALP  hsa-OCN  hsa-RUNX2  hsa-P16  hsa-P21  hsa-PPARG  hsa-FABP4  hsa-VEGF  hsa-ANG1  hsa-miR-146a-5p | Forward Reverse Forward Reverse Forward Reverse Forward Reverse Forward Reverse Forward Reverse Forward Reverse Forward Reverse Forward Reverse Forward Reverse | GTCTCCTCTGACTTCAACAGCG ACCACCCTGTTGCTGTAGCCAA GGTCAGGTTTCAACAGCCCTAG GCTCATTCCGATTGTCGTGGAG CGCTACCTGTATCAATGGCTGG CTCCTGAAAGCCGATGTGGTCA CCCAGTATGAGAGTAGGTGTCC GGGTAAGACTGGTCATAGGACC CTCGTGCTGATGCTACTGAGGA GGTCGGCGCAGTTGGGCTCC AGGTGGACCTGGAGACTCTCAG TCCTCTTGGAGAAGATCAGCCG AGCCTGCGAAAGCCTTTTGGTG GGCTTCACATTCAGCAAACCTGG ACGAGAGGATGATAAACTGGTGG GCGAACTTCAGTCCAGGTCAAC TTGCCTTGCTGCTCTACCTCCA GATGGCAGTAGCTGCGCTGATA CAACAGTGTCCTTCAGAAGCAGC CCAGCTTGATATACATCTGCACAG UGAGAACUGAAUUCCAUGGGUU |

**Table S4.** Primer sequences used for qRT-PCR in this study.
